# Depression Levels Are Associated with Reduced Capacity to Learn to Actively Avoid Aversive Events in Young Adults

**DOI:** 10.1523/ENEURO.0034-25.2025

**Published:** 2025-09-09

**Authors:** Ryan J. Tomm, Brandon J. Forys, Liz Kalenteridis, Ian D. Daly, Alex R. Terpstra, Luke Clark, Stan B. Floresco, Trisha Chakrabarty, Rebecca M. Todd

**Affiliations:** ^1^Department of Psychology, University of British Columbia, Vancouver, British Columbia V6T 1Z4, Canada; ^2^Djavad Mowafaghian Centre for Brain Health, University of British Columbia, Vancouver, British Columbia V6T 1Z3, Canada; ^3^Department of Psychiatry, University of British Columbia, Vancouver, British Columbia V6T 2A1, Canada

**Keywords:** avoidance, Beck Depression Inventory, depression, dimensional approaches, effort cost, translational research

## Abstract

Depression and anxiety are often characterized by altered reward-seeking and avoidance, respectively. Yet less is known about the relationship between depressive symptoms and specific avoidance behaviors. To address this gap, we conducted two studies. In Study 1, undergraduates and online workers completed an uninstructed go/no-go avoidance task (*N*_Total_ = 465) as a reverse translation of a rodent paradigm. Participants exhibited a wide range of symptom scores on the Beck Depression Inventory-II (BDI-II), ranging from low to severe. In Study 1, cues were used to signal the response type (go/active vs no-go/inhibitory) required to avoid an aversive sound. Higher depressive scores were associated with poorer acquisition of active avoidance in undergraduates. Overall participants showed lower accuracy for active than inhibitory avoidance. To examine whether the better no-go trial performance reflected a prepotent response to avoid aversive outcomes, in Study 2, undergraduates (*N*_Total_ = 330) completed a version of the task that included reward-seeking. Here all participants showed higher accuracy for active reward-seeking and inhibitory avoidance, consistent with a prepotent response to inhibit action to avoid aversive consequences. These findings suggest that in young adults, depressive symptoms are associated with difficulty in overriding prepotent responses to actively avoid aversive outcomes in the absence of reward. This work bridges the gap between preclinical animal models and clinical research, offering insights that could guide the development of more targeted clinical interventions.

## Significance Statement

Translational studies in community samples are crucial for bridging the gap between rodent models, which delineate neural circuitry and pharmacology underlying specific behaviors, and the presentation of mood disorders in clinic settings. Building on rodent studies of avoidance behaviors, thought to be linked to depression, this study examines how depressive symptom scores relate to specific types of avoidance. Our findings revealed that higher depressive symptom scores were associated with reduced capacity to learn active avoidance behaviors, which involved overriding a prepotent response to inhibit action to avoid aversive consequences. This work bridges the gap between preclinical animal models and clinical research, offering insights that may guide the development of more targeted clinical interventions.

## Introduction

Stimuli that predict aversive events typically evoke avoidance responses aimed at minimizing anticipated threats. Depending on the situation, an active strategy, such as taking an action (walking away), may be most effective, while in other situations, the inhibition of motor output (staying put to avoid detection) may be the more prudent strategy. Although effective in many contexts, these strategies can become maladaptive in depression and anxiety, interfering with goal-directed behavior (Ottenbreit et al., 2014; Haskell et al., 2020).

Depression is a leading cause of global disability (Whiteford et al., 2013; World Health Organization, 2017), yet its cognitive and behavioral mechanisms remains to be fully understood. The Altered Computations underlying Decision Making (ACDM) framework posits that decision-making biases perpetuate both depression and anxiety (Bishop and Gagne, 2018). Depression is marked by reduced engagement in reward-seeking, while anxiety by heightened avoidance. In depression, impairments arise from underestimating the probability and value of positive outcomes, and overestimating the effort required to obtain them (Bishop and Gagne, 2018), ultimately leading to reduced engagement in actions. Supporting this view, individuals with major depressive disorder (MDD) choose high-effort, high-reward options less frequently, anticipate fewer positive experiences, and rate them as less pleasurable (MacLeod and Salaminiou, 2001; Treadway et al., 2012; Mukherjee et al., 2020; Horne et al., 2021). Although the ACDM primarily distinguishes between depression-related biases in reward-seeking, it also suggests that effort-related impairments may extend to avoidance contexts and contribute to reduced active avoidance.

Despite this, the role of active versus inhibitory forms of avoidance remains underexplored in depression, reflecting broader trends in which negatively valenced systems are predominantly studied in anxiety (Craske et al., 2009). Cognitive theories of depression emphasize a negativity bias in attention, memory, and future expectations (Mogg et al., 2006; Fales et al., 2008; Disner et al., 2011) but often rely on self-report and lack emphasis on behaviors with translational utility for identifying cross-species neurobiological mechanisms.

Reinforcement learning tasks offer a translational approach for examining negatively valenced systems and have been applied across neuropsychiatric conditions (Endrass et al., 2011; Palminteri et al., 2012; Reinen et al., 2016; Waltz et al., 2018), including depression (Chase et al., 2010; Robinson et al., 2012; Mkrtchian et al., 2017; Mukherjee et al., 2020; Smith et al., 2023). These studies typically involve probabilistic and reversal learning tasks to probe sensitivity to reward and punishment. Findings remain mixed: some report reward-specific impairments in depression (Robinson et al., 2012), others find broader impairments across valence (Chase et al., 2010; Mkrtchian et al., 2017; Mukherjee et al., 2020) or even heightened punishment sensitivity (Murphy et al., 2003; Nord et al., 2018), while others identify learning-specific impairments (Chase et al., 2010; Mukherjee et al., 2020). These inconsistencies highlight the need for behavioral assays that isolate avoidance processes and align with cross-species models.

Translational gaps can stem from task design. Human studies typically use secondary reinforcers (i.e., monetary rewards or feedback), with punishment operationalized as monetary loss, and avoidance inferred from decreased selection of high-loss options, often omitting safety signals. In contrast, animal paradigms use primary reinforcers (i.e., shock) and deterministic contingencies and explicitly distinguish between active and inhibitory avoidance (Piantadosi et al., 2018; Capuzzo and Floresco, 2020). Although functional magnetic resonance imaging (fMRI) studies show overlapping blood-oxygenation-level-dependent (BOLD) responses to primary and secondary aversive cues, regions like the amygdala are more responsive to primary aversive cues (Delgado et al., 2011). Importantly, shared BOLD activation does not necessarily imply equivalent neural mechanisms—especially when task features might differ meaningfully. Translating animal behavioral paradigms to humans has been proposed as a promising strategy to enhance cross-species translation and improve psychiatric treatment development (Kirlic et al., 2017).

To address these gaps, we adapted a validated rodent task designed to assess both active and inhibitory avoidance (Piantadosi et al., 2018; Capuzzo and Floresco, 2020) and deployed it in a large online sample. While prior human studies have included related features, our avoidance task was modeled to parallel the original rodent paradigm. Our aim was to examine how depressive symptom severity relates to the ability to learn and flexibly implement active and inhibitory avoidance strategies. Using a dimensional approach aligned with Research Domain Criteria (RDoC) principles, we recruited a nonclinical sample reporting a broad range of depressive symptoms. We hypothesized that higher depressive symptom scores would be associated with impairments in active—but not inhibitory—avoidance, consistent with ACDM predictions of reduced behavioral engagement stemming from effort overestimation.

## Materials and Methods

### Study 1 (avoidance)

#### Participants

We conducted a power analysis using G*Power to detect a small effect size $\left(\;f^{2}=0.02\right)$, indicating that a sample size of *N* = 395 was required to achieve 80% power at *α* = 0.05. To account for the higher attrition rates typically observed in online studies, we recruited additional participants. Undergraduates (*N*_Undergraduates _= 475) and online workers (*N*_OnlineWorkers _= 292; recruited via Prolific; www.prolific.co) consented to perform an active/inhibitory avoidance task (*N*_Total _= 767). Undergraduates from the University of British Columbia Psychology Human Subjects Pool were compensated a 1% point increase in their course grade; Prolific workers were compensated £10.59/h. The online avoidance task was unsupervised and uninstructed to allow for instrumental learning processes. Participants were excluded for several reasons, including failure to complete the pre-task survey, failure of survey attention checks, failure to reach criterion accuracy during acquisition, obtaining a *d*’ < 0.50 during the intermixed task stage, or failure to complete the task. After cleaning, *N*_Total _= 465 (*N*_Undergraduates _= 278; *N*_OnlineWorkers _= 187) were included in the analyses. For details on participant exclusion rates, see Discussion and Extended Data (Extended Data Table 1-1). The study was approved by the University of British Columbia Behavioral Research Ethics Board (BREB) under certificate H20-01388. Demographic information can be found in Table 1.

#### Materials

##### Stimuli

The task was created in PsychoPy 2020.1 (RRID: SCR_006571) and distributed via Pavlovia (www.pavlovia.org; Peirce et al., 2019). Simple shapes signaled the type of response (active vs inhibitory) required to avoid an aversive sound. Coauthor I.D.D. recorded a set of screeching and scraping sounds (i.e., knife on glass, fork on plate, metal on slate), from which 45 were pilot-tested for unpleasantness and salience (*N* = 45). Using 9-point Likert scales, eight sounds with the highest combined ratings (unpleasantness: *M* = 6.87–7.57; salience: *M* = 5.82–6.83) and lowest variance were selected. These eight aversive sounds were randomly presented on failed trials and were found to be highly motivating. In Study 1, 92.46% of participants who responded to a debriefing question (*N* = 464) endorsed the aversive sounds as motivating to avoid. Rapid acquisition of instrumental avoidance responses further supports the functional aversiveness of the stimuli. On successful trials, a white border around the gray background signaled safety.

##### Measures

Depressive and anxiety symptom scores were derived from the clinically validated Beck Depression Inventory-II (BDI-II; Beck et al., 1988b; Beck et al., 1996) and the Beck Anxiety Inventory (BAI; Beck et al., 1988a), respectively. One question (suicidality ideation) was removed from the BDI-II for ethical considerations. BDI-II symptom scores were calculated as a proportion score (BDI-II_score_/BDI-II_max_possible_score_) for each participant. Similarly, BAI scores are reported as proportion scores (BAI_score_/BAI_max_possible_score_) for consistency and comparability.

#### Procedure

Participants were tested on a computer-based avoidance task that was reverse-translated from a rodent operant paradigm assessing active/inhibitory avoidance—with some modifications (Piantadosi et al., 2018; Capuzzo and Floresco, 2020). Prior to the avoidance task, participants completed an effort calibration and a volume calibration to control for differences in physical ability and computer systems (see https://osf.io/5sepm/).

##### Active/inhibitory avoidance task

**Figure 1.**
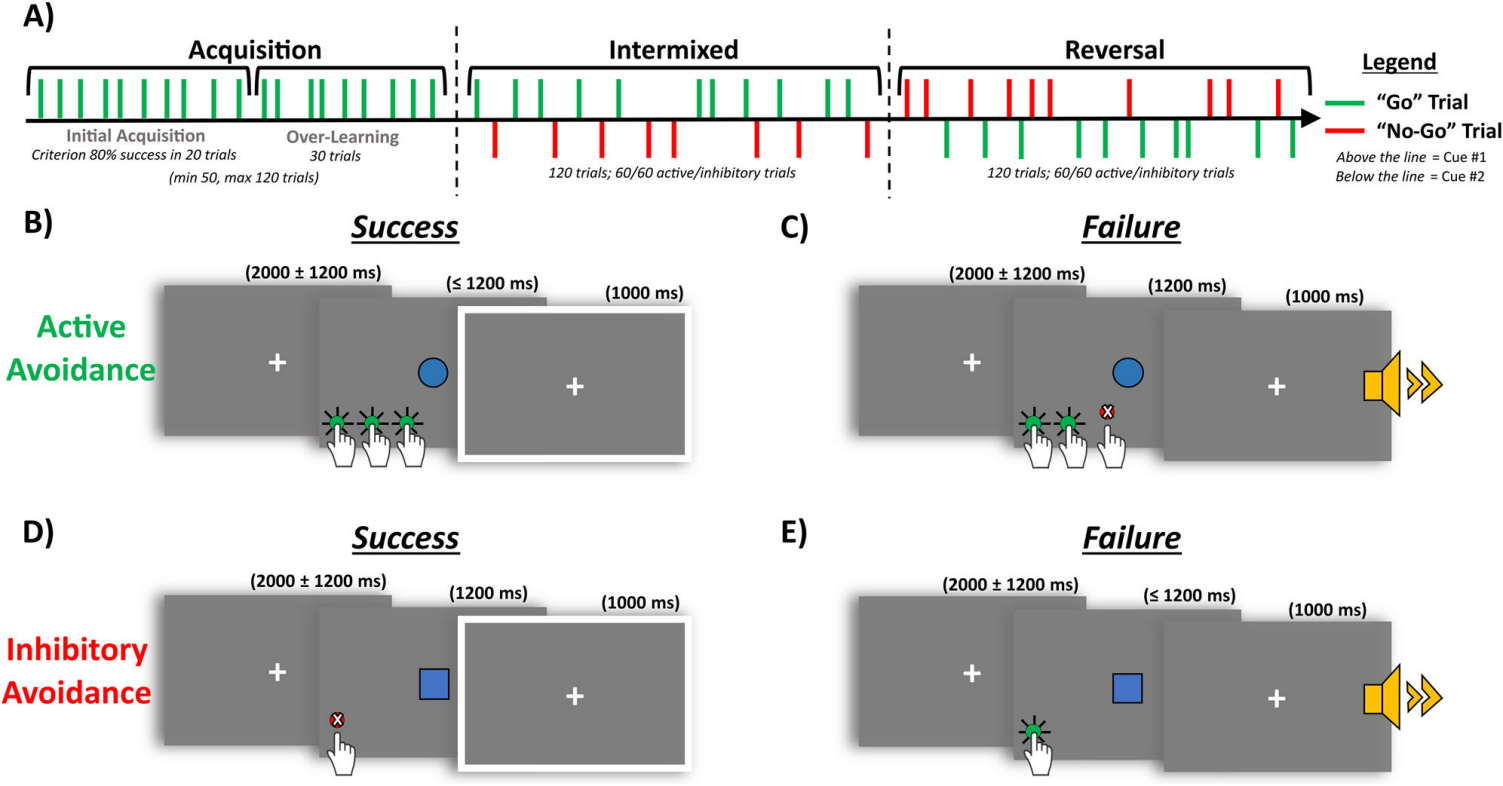
Study 1: Active/inhibitory avoidance task. ***A***, Schematic of task stages: acquisition, intermixed, and reversal. Green lines represent active avoidance (“Go”) trials, while red lines represent inhibitory avoidance (“No-Go”) trials. The black arrow indicates time progression. During acquisition, participants were required to reach a criterion of 80% correct active avoidance trials over a 20-trial period (i.e., initial acquisition) or complete a maximum of 120 trials. Upon reaching criterion, participants completed 30 over-learning trials, followed by an unsignaled transition into the intermixed stage. In this stage, participants flexibly alternated between active and inhibitory avoidance responses. The reversal stage followed another unsignaled transition, during which the cues signaling active and inhibitory avoidance responses are reversed. ***B***, Successful active avoidance: A blue circle signals an active avoidance trial. The participant makes sufficient keyboard presses (green circles) within 1,200 ms cue period, avoiding an aversive sound, as indicated by the safety signal (white border). ***C***, Failed active avoidance: A blue circle signals an active avoidance trial, but insufficient or no keyboard presses (red circle) were made within the 1,200 ms, resulting in an aversive sound (yellow speaker). ***D***, Successful inhibitory avoidance: A blue square signals an inhibitory avoidance trial. The participant withholds keyboard presses for the full 1,200 ms, avoiding an aversive sound, indicated by the safety signal. ***E***, Failed inhibitory avoidance: A blue square signals an inhibitory avoidance trial, but the participant fails to withhold keyboard presses within the 1,200 ms, resulting in an aversive sound.

Specific shapes (circle or squares; counterbalanced) signaled active (“Go”) versus inhibitory (“No-Go”) responses required to avoid a highly aversive sound. The avoidance task consisted of three task stages: acquisition, intermixed, and reversal (Fig. 1*A*). (1) The acquisition stage required learning an active avoidance response. During the acquisition stage, participants had to reach a criterion performance of 80% successful trials within the previous 20 trials (maximum 120 trials). Once acquisition criterion was reached, participants performed an additional 30 “over-learning” active avoidance trials before an unsignaled transition into the next task stage. Participants failing to reach criterion performance during the acquisition stage were excluded from data analysis. (2) The intermixed stage required participants to learn the inhibitory avoidance response while flexibly deploying both active and inhibitory responses. This stage consisted of 120 avoidance trials (60 active, 60 inhibitory), presented in a pseudorandomized order. (3) The reversal stage also consisted of 120 avoidance trials (60 active, 60 inhibitory; pseudorandomized), but with active and inhibitory response contingencies reversed. The multiple task stages allowed us to assess distinct patterns in the acquisition and expression of active/inhibitory avoidance, as well as reversal learning. Importantly, participants were not instructed about the cue–response contingencies to allow the acquisition through reinforcement, to mirror the rodent paradigm the task was translated from.

Trials began with a fixation cross onscreen (ISI; 2,000 ms; jittered 1,200 ms). Active avoidance required an effortful response—specifically, 3, 4, or 5 rapid button presses (criterion determined during effort calibration test). On successful active trials (Fig. 1*B*), participants made the required response within the cue period (≤1,200 ms), resulting in the avoidance of the aversive sound and the presentation of a safety signal (1,000 ms). On failed active trials (Fig. 1*C*), either an insufficient response or no response within the cue period triggered the aversive sound (1,000 ms). On inhibitory trials, participants were required to withhold responding. On successful inhibitory trials (Fig. 1*D*), participants made no button presses during the cue period (1,200 ms), resulting in the avoidance of the aversive sound and the presentation of a safety signal (1,000 ms). On failed inhibitory trials (Fig. 1*E*), an erroneous button press triggered the aversive sound. A graphical overview of the avoidance task is provided in Figure 1.

#### Statistical analysis

All analyses were conducted in R 4.2.1 (R Core Team, 2013) using RStudio (Booth et al., 2018). Primary outcome measures included proportion correct for active and inhibitory trials across task stages and the number of trials to criterion during acquisition. Within-subjects ANOVAs were used except where otherwise stated. Significant main effects or interactions were followed by pairwise comparisons using the *emmeans* package (Searle et al., 1980; Lenth, 2017), with Tukey's honest significant difference (HSD) correction. Between-subject ANOVAs tested sex and sample effects on BDI-II scores. To examine individual differences, we used regression and linear mixed models (lmerTest; fit by REML, *t* tests using Satterthwaite's method; Bates et al., 2015; Kuznetsova et al., 2017) to assess BDI-II scores effects on task performance. To account for multiple comparisons, the Benjamini–Hochberg false discovery rate (FDR) correction was applied (Benjamini and Hochberg, 1995). We present only the BDI-II analyses in the main text, while corresponding analyses for BAI scores are presented in Extended Data (Extended Data Figs. 2-1, 4-1; Extended Data Tables 3-1, 3-3).

### Study 2 (reward-seeking/avoidance)

#### Participants

Undergraduate participants (*N* = 771) from the University of British Columbia Psychology Human Subjects Pool were recruited to perform a reward-seeking/avoidance task. Power analysis procedures were identical to Study 1. Recruitment focused exclusively on undergraduates, as effects in Study 1 were strongest in this population. Compensation was identical to the undergraduate sample in Study 1. To motivate performance during reward-seeking trials, participants were told their accumulated points would contribute to the value of a gift card, although participants ultimately received a $5 gift card regardless of performance. Exclusion criteria were similar to Study 1, with the added requirement that participants reach criterion accuracy during acquisition for both reward-seeking and avoidance trials independently. Because the task was designed as a reinforcement-based learning paradigm—with minimal instructions, no explicit information about contingencies, and no practice trials—and given variability in motivation among undergraduates completing online studies for credit, exclusions rates were higher than expected, resulting in lower-than-ideal power. For details on participant exclusion rates, see Discussion and Extended Data (Extended Data Table 1-1). After cleaning, the final sample included *N* = 330 participants (*N*_female_ = 245; *N*_male_ = 85). The study was approved by the University of British Columbia Behavioral Research Ethics Board (BREB) under certificate H20-01388. Demographic information for Study 2 can be found in Table 1.

**Table 1.** Demographic information for all participants

Sample	*N*	*N*_Female_	*N*_Male_	*M*_Age_	SD_Age_	Range_Age_
Study 1: Avoidance						
Undergraduates	278	226	52	20.81	3.81	17–53
Online Workers	187	54	133	27.74	4.77	19–37
Total	465	280	185	23.60	5.42	17–53
Study 2: Reward-Seeking/Avoidance						
Undergraduates	330	245	85	20.71	3.58	17–54

For details on exclusions, see Extended Data Table 1-1.

10.1523/ENEURO.0034-25.2025.t1-1 Table 1-1 Download Table 1-1, TEX file.

#### Materials

##### Stimuli and measures

The task was implemented using PsychoPy and Pavlovia (same as Study 1) with modification to incorporate reward-seeking trials. Stimuli included four simple shapes (blue; square, circle, triangle, hexagon) counterbalanced across response type (active vs inhibitory) and motivational context (reward-seeking vs avoidance). As in Study 1, participants completed questionnaire measures, effort, and volume calibrations procedures.

##### Mixed-motivation go/no-go task

The mixed-motivation task consisted of two stages: (1) an acquisition stage, where participants learned active reward-seeking and active avoidance responses, and (2) an intermixed stage, which required the flexible expression of active and inhibitory responses across reward-seeking and avoidance contexts. During the acquisition stage, participants had to reach 80% accuracy within the previous 20 trials, independently for both active reward-seeking and active avoidance trials. After reaching the acquisition criterion, participants completed 24 “over-learning” trials (12 reward-seeking and 12 avoidance) before an unsignaled transition into the intermixed stage. Participants who failed to reach criterion were excluded from analysis. The intermixed stage consisted of 240 trials (60 of each type—active reward-seeking, inhibitory reward-seeking, active avoidance, inhibitory avoidance), presented in a pseudorandomized order. The reversal stage used in Study 1 was omitted.

Trials began with a fixation cross (ISI; 2,000 ms; jittered 1,200 ms). Active responses required 3, 4, or 5 button presses within the 1,200 ms cue period (threshold determined during effort calibration). On successful trials, participants either earned 5 points or avoided an aversive sound, depending on the motivational context. Successful reward-seeking trials provided a reward signal (1,000 ms; white border), while successful avoidance trials were followed by a safety signal (1,000 ms; white border). On failed trials, participants either received no points (reward-seeking) or were presented with an aversive sound (avoidance). Points accumulated were displayed on reward-seeking trials, and a musical tone (C major chord; 1,100 ms) played each time participants earned an additional 25 points.

#### Statistical analysis

Analytical procedures followed Study 1. Accuracy (proportion correct) and trials to criterion during acquisition were the primary outcomes. Linear mixed models tested BDI-II symptom scores effects on active and inhibitory accuracy across motivational contexts. FDR corrections were used for multiple comparisons.

### Code accessibility

No computational neuroscience models were developed for this study. However, extended data and code used to conduct the linear mixed models are available at https://osf.io/5sepm/.

## Results

### Study 1 (avoidance)

#### Demographics

**Figure 2.**
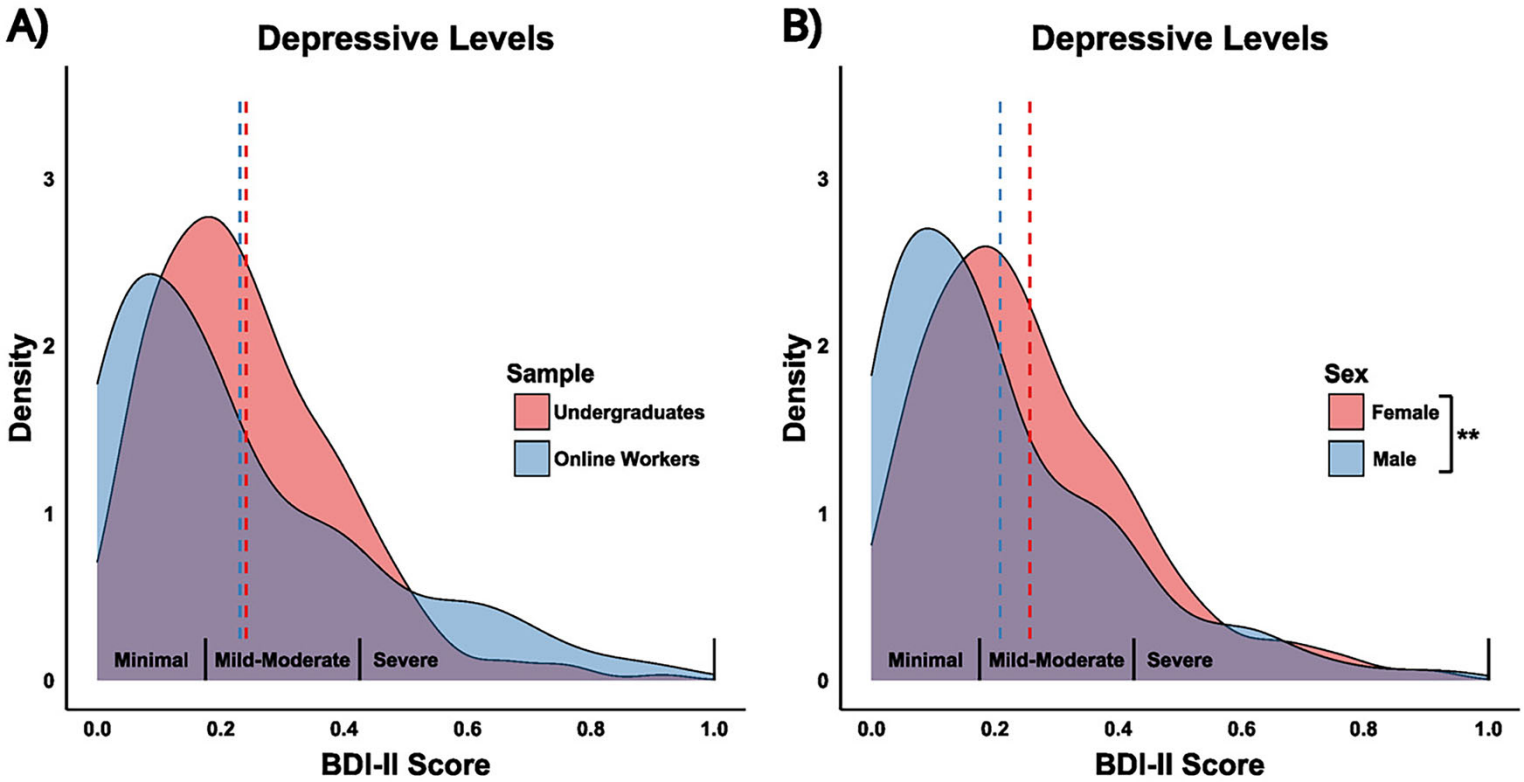
Study 1: Distribution of depressive symptom scores across samples and sexes. Density plots representing the distribution of Beck Depression Inventory-II (BDI-II) scores. ***A***, Depressive symptom score distributions by sample: Undergraduates (red) and Online Workers (blue). ***B***, Depressive symptom score distributions by sex: Female (red) and Male (blue). The *x*-axis represents proportion scores, where raw BDI-II symptom scores, ranging from 0 to 60, have been divided by the maximum possible score (60) to produce a proportion between 0 and 1. This adjustment was made because the suicide ideation question was removed for ethical consideration. The labels on the *x*-axis—Minimal (0–13), Mild-Moderate (14–28), Severe (29–63)—reflect typical ranges of raw symptom scores for ease of interpretation. Dashed vertical lines represent the mean BDI-II symptom score for each group. In panel ***B***, a significant difference in depressive levels between sexes is indicated (*p* < 0.01), with females scoring higher on average than males. See Extended Data Figures 2-1 (BAI distributions) and 2-2 (Age distributions).

To assess differences between undergraduates and online workers, we first compared self-reported depressive and anxiety symptom scores. There was no difference in depressive symptom scores (*F*_(1,463)_ = 0.34, *p* = 0.56; Fig. 2*A*), but undergraduates reported significantly higher anxiety symptom scores compared with online workers (*F*_(1,463)_ = 13.84, *p* < 0.001; Extended Data Fig. 2-1). Sex and gender responses were highly congruent (>96%); due to limited statistical power for non-cis gender categories, subsequent analyses refer to sex only. Females reported higher depressive (*F*_(1,463)_ = 7.96, *p* < 0.01; Fig. 2*B*) and higher anxiety (*F*_(1,463)_ = 33.63, *p* < 0.001; Extended Data Fig. 2-1) symptom scores than males. Finally, there was a significant age difference between samples (*F*_(1,462)_ = 299.8, *p* < 0.001), with undergraduates being younger on average compared with online workers (Extended Data Fig. 2-2).

10.1523/ENEURO.0034-25.2025.f2-1 Figure 2-1 **Study 1: Distribution of Anxiety Scores Across Samples and Sexes.** Density plots representing the distribution of Beck Anxiety Inventory (BAI) scores. **A)** Anxiety score distributions by sample: Undergraduates (red) and Online Workers (blue). **B)** Anxiety score distributions by sex: Female (red) and Male (blue). The x-axis represents proportion scores, where raw BAI scores, ranging from 0-63, have been divided by the maximum possible score (63) to produce a proportion between 0 and 1. This adjustment was made for comparability between the BDI-II and BAI scales. The labels on the x-axis -- Minimal (0-7), Mild-Moderate (8-25), Severe (26-63) -- reflect typical ranges of raw scores for ease of interpretation. Dashed vertical lines represent the mean BAI score for each group. In panel A, a significant difference in anxiety levels between sample groups is indicated (*p* < .001), with undergraduates scoring higher on average than online workers. In panel B, a significant difference in anxiety levels between sexes is indicated (*p* < .001), with females scoring higher on average than males. Download Figure 2-1, TIF file.

10.1523/ENEURO.0034-25.2025.f2-2 Figure 2-2 **Study 1: Age Distribution Across Samples.** Density plot representing the distribution of ages for undergraduates (red) and online workers (blue). Dashed vertical lines represent the mean age for each group. A significant difference in age between the samples are indicated (*p* < .001), with online workers being older on average compared to undergraduates. Download Figure 2-2, TIF file.

#### Avoidance task

##### Within-subject results

**Figure 3.**
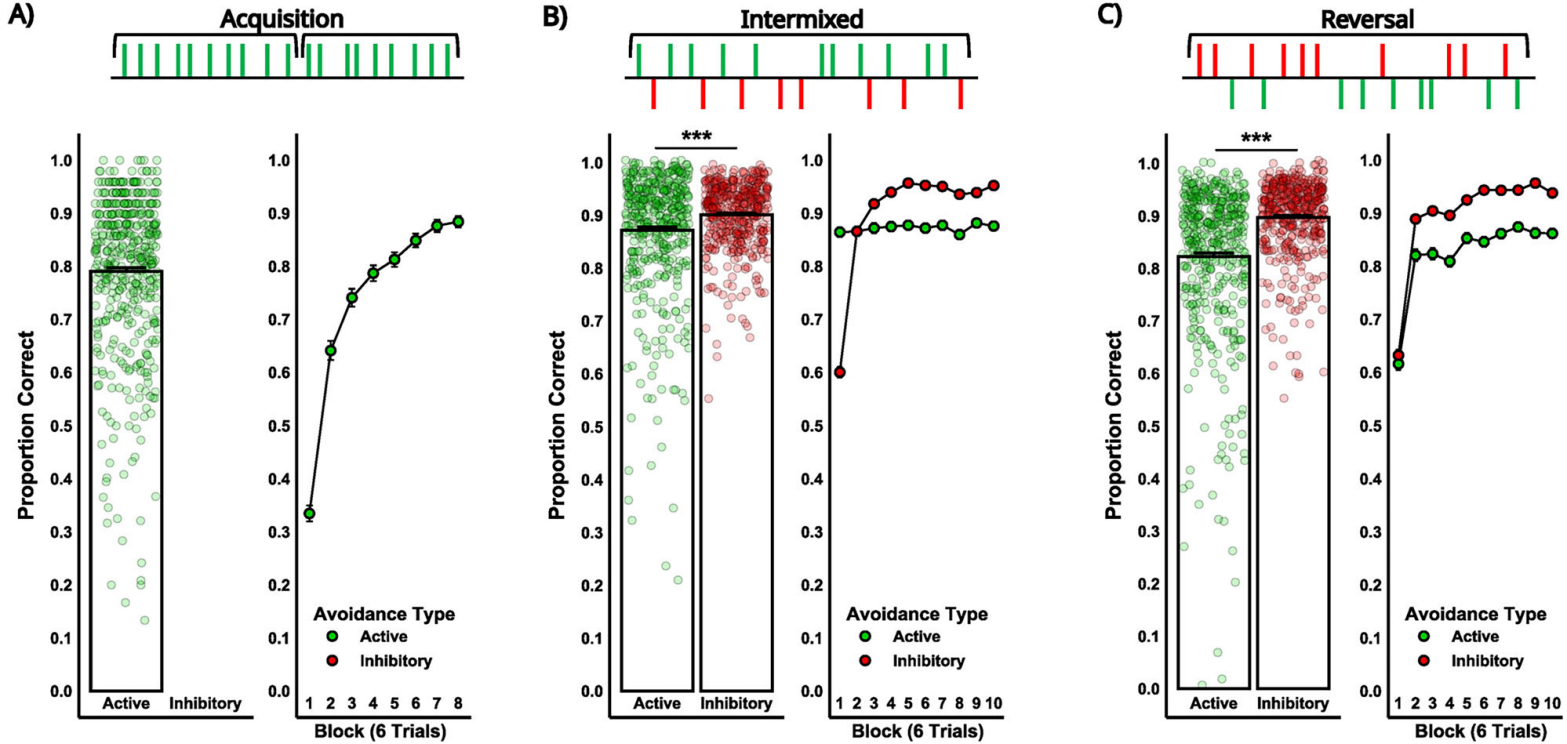
Study 1: Avoidance performance across task stages. Proportion correct for active (green) and inhibitory (red) avoidance responses across three task stages: ***A***, Acquisition; ***B***, intermixed, and ***C***, reversal. The left panels display overall accuracy, with circles representing individual participant performance for active and inhibitory avoidance, respectively. The right panels show accuracy across blocks of six trials, with circles representing mean performance for each avoidance type. Proportion correct reflects the ratio of successful avoidance responses relative to the total number of trials for each avoidance type. Significant differences between active and inhibitory avoidance during the intermixed and reversal stages are indicated (*p* < 0.001).

**Figure 4.**
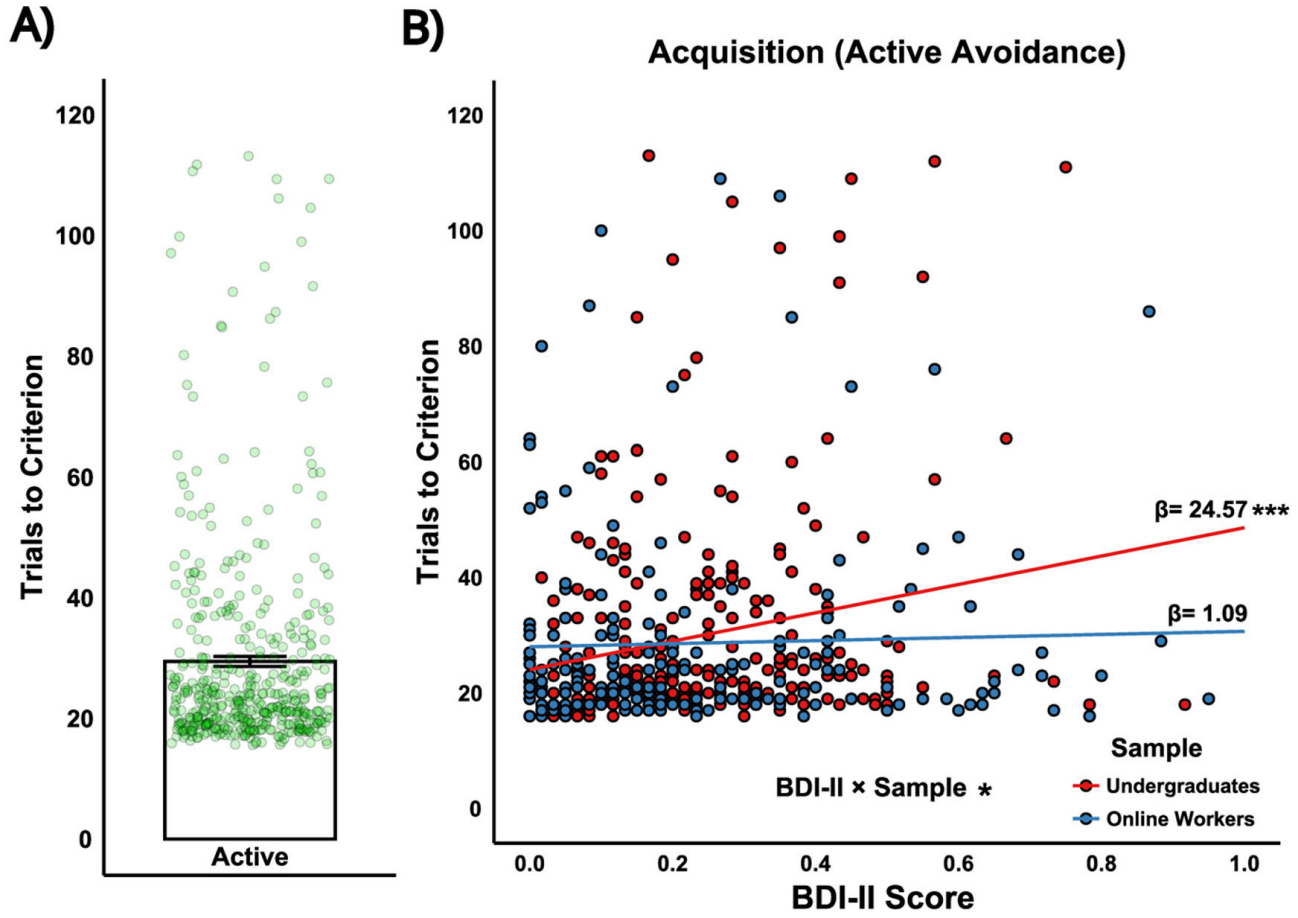
Study 1: Trials to criterion for active avoidance during the acquisition stage. ***A***, Number of trials required to reach criterion for all participants in the avoidance task. Individual data points (green circles) represent the number of trials each participant required to reach the criterion of 80% correct responses within 20-trial period during acquisition. ***B***, Interaction between depressive symptom scores (BDI-II proportion scores) and the sample group (Undergraduates vs Online Workers) predicting trials to criterion for active avoidance. The regression lines show the relationship between depressive symptom scores and trials to criterion for each sample, with a stronger effect observed in undergraduates (*β* = 24.57) compared with online workers (*β* = 1.09). A significant main effect of BDI-II symptom scores (*p* < 0.001) and a significant BDI-II × Sample interaction (*p* < 0.05) are indicated. See Extended Data Figure 4-1 for corresponding BAI effects.

*Acquisition*. The acquisition task stage assessed initial learning of the active avoidance response. Participants showed robust acquisition, with an average accuracy of 0.79 (SD = 0.15; Fig. 3*A*). The mean number of trials to reach criterion (≥80% correct in 20 trial period) was 29.65 (SD = 18.42; range, 16–120 trials; Fig. 4*A*). Higher BDI-II scores were associated with a greater number of trials needed to reach criterion during acquisition, but this effect was specific to undergraduates (Fig. 4*B*; see https://osf.io/5sepm/).

10.1523/ENEURO.0034-25.2025.f4-1 Figure 4-1 **Study 1: Trials to Criterion for Active Avoidance During the Acquisition Stage**. **A)** Number of trials required to reach criterion for all participants in the avoidance task. Individual data points (green circles) represent the number of trials each participant required to reach the criterion of 80% correct responses within 20-trial period during acquisition. **B)** Interaction between anxiety scores (BAI proportion scores) and the sample group (Undergraduates vs. Online Workers) predicting trials to criterion for active avoidance. The regression lines show the relationship between anxiety scores and trials to criterion for each sample, with a stronger effect observed in undergraduates (*β* = 16.14) compared to online workers (*β* = -3.81). A significant main effect of BAI scores (*p* < .01) and a significant BAI × Sample interaction (*p* < .05) are indicated. Download Figure 4-1, TIF file.

*Intermixed and reversal*. The intermixed task stage assessed participants’ ability to switch between active and inhibitory responses using discriminative cues, while the reversal task stage assessed behavioral flexibility when cue–response contingencies were reversed. A 2 × 2 within-subjects ANOVA assessing proportion correct, with Avoidance Type (active, inhibitory) and Task Stage (intermixed, reversal) as within-subjects factors, revealed a significant effect of Avoidance Type and Task Stage, and a significant interaction (Table 2). Follow-up analysis revealed higher accuracy on inhibitory compared with active trials in both the intermixed (*M*_Inhibitory_ = 0.90, SD = 0.06; *M*_Active_ = 0.87, SD = 0.12; *t*_(711)_ = −4.74, *p* < 0.001; Fig. 3*B*) and reversal stages (*M*_Inhibitory_ = 0.90, SD = 0.07; *M*_Active_ = 0.82, SD = 0.15; *t*_(711)_ = −12.18, *p* < 0.001; Fig. 3*C*). Accuracy on active trials was also higher in the intermixed compared with the reversal stage (*t*_(877)_ = 10.49, *p* < 0.001), whereas inhibitory accuracy did not differ by stage (*t*_(877)_ = 0.81, *p* = 0.42).

**Table 2.** Study 1—2 × 2 within-subjects ANOVA table for active and inhibitory avoidance accuracy for intermixed and reversal task stages

Proportion Correct ∼ Avoidance Type × Task Stage + Error (Participant/(Avoidance Type × Task Stage))						
Effect	df	Sum Sq	Mean Sq	*F* value	*p* value	*η*^2^ (partial)
Within-Subjects						
Avoidance Type***	1	1.241	1.241	92.32	<0.001	0.17
Residuals (Avoidance Type)	464	6.239	0.013			
Task Stage***	1	0.327	0.327	51.38	<0.001	0.10
Residuals (Task Stage)	464	2.950	0.006			
Avoidance Type × Task Stage***	1	0.240	0.240	61.78	<0.001	0.12
Residuals (Avoidance Type × Task Stage)	464	1.801	0.004			
Participant						
Residuals	464	10.620	0.023			

*** *p* < 0.001.

##### Between-subject results

**Figure 5.**
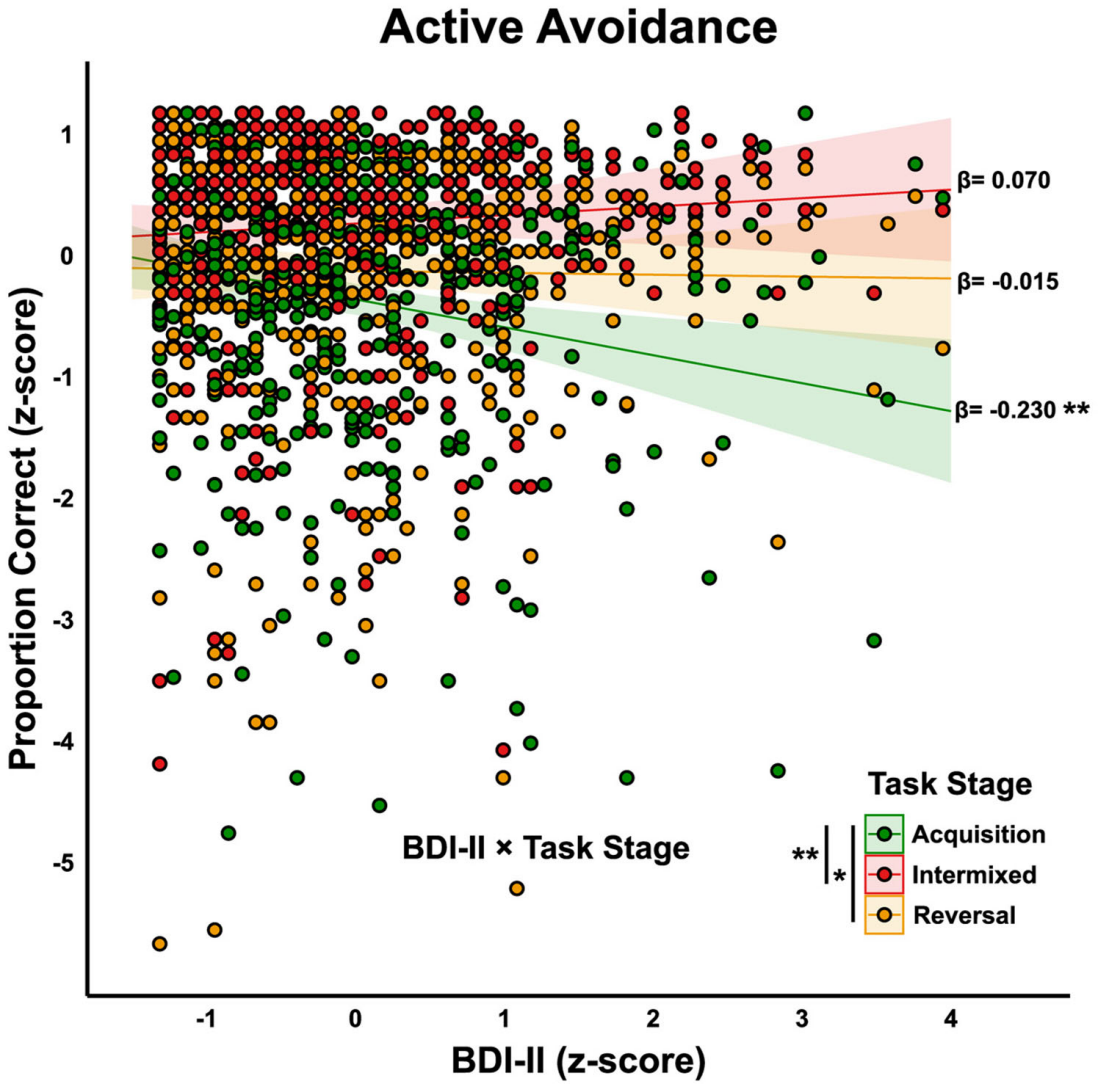
Study 1: Linear mixed model predicting active avoidance accuracy: interaction between BDI-II scores and task stage. *Z*-normalized depressive symptom scores (BDI-II) interacted with task stage (acquisition, intermixed, reversal) to predict active avoidance accuracy (proportion correct; *z*-normalized). Formula: Accuracy* ∼ *BDI-II* × *Sex* × *Task Stage* × *Sample* + *(*1|* Participant). Each circle represents individual participant data. Colored regression lines show the relationship between depressive scores and active accuracy across task stages: acquisition (green), intermixed (red), and reversal (amber). A significant negative relationship between BDI-II scores and accuracy in the acquisition stage (*β* = −0.230), while this relationship was attenuated in the intermixed (*β* = −0.015), and reversal (*β* = 0.070) stages. Significant BDI-II × Task Stage interactions are indicated. Asterisks next to regression lines denotes slopes significantly different from zero; asterisks between task stages indicate significant differences in the slopes between levels. Statistical significance denoted as follows: *p < 0.05, **p < 0.01. Full model results are presented in Table 3.

*Depressive symptom scores and active avoidance accuracy*. To examine the relationship between depressive symptom scores and active avoidance accuracy, we used a linear mixed model with BDI-II symptom scores (*z*-normalized, grand-mean centered) as the primary predictor. The model included Sex (female, male), Task Stage (acquisition, intermixed, reversal), and Sample (undergraduates, online workers) as fixed effects and Participant as a random intercept. Proportion correct on active trials was also *z*-normalized (grand-mean centered). To ensure model stability, we adopted a simplified random-effects structure that excluded a random slope for Task Stage. As shown in Table 3, the model revealed a significant main effect of BDI-II (*β* = −0.230, SE = 0.074, *p* = 0.009), indicating that higher depressive symptoms were associated with lower active avoidance accuracy when all other variables were at their reference levels (i.e., female, acquisition, undergraduates). After controlling for multiple comparisons, there were no significant interactions between BDI-II and Sex or Sample. However, we observed a significant interaction between BDI-II and Task Stage, with the relationship between depressive symptoms and active avoidance accuracy changing in the intermixed (*β* = 0.300, SE = 0.083, *p* = 0.003) and reversal stages (*β* = 0.215, SE = 0.083, *p* = 0.041), relative to acquisition. These interactions suggest that the negative relationship between depressive symptoms and active avoidance accuracy was strongest during initial learning (acquisition) and was attenuated at later stages when avoidance responses are well-learned or inhibitory control was required (Fig. 5).

**Table 3.** Study 1—linear mixed model for BDI-II predicting active avoidance accuracy

Active avoidance accuracy ∼ BDI-II (prop. score) × Sex × Task Stage × Sample + (1 | Participant)					
Fixed Effects	*β* (SE)	df	*t* value	*p* value	*p* adj
Intercept	−0.361 (0.065)	1,076.704	−5.572	<0.001	<0.001
BDI-II (prop. score)**	−0.230 (0.074)	1,076.704	−3.101	0.002	0.009
Sex (Male)	0.279 (0.150)	1,076.704	1.857	0.064	0.169
Task Stage (Intermixed)***	0.624 (0.073)	914	8.583	<0.001	<0.001
Task Stage (Reversal)**	0.234 (0.073)	914	3.214	0.001	0.008
Sample (Online Workers)	0.111 (0.152)	1,076.704	0.731	0.465	0.667
BDI-II × Sex (Male)	0.037 (0.205)	1,076.704	0.179	0.858	0.895
BDI-II × Task Stage (Intermixed)**	0.300 (0.083)	914	3.599	<0.001	0.003
BDI-II × Task Stage (Reversal)*	0.215 (0.083)	914	2.576	0.010	0.041
Sex (Male) × Task Stage (Intermixed)	−0.147 (0.169)	914	−0.872	0.383	0.645
Sex (Male) × Task Stage (Reversal)	−0.203 (0.169)	914	−1.205	0.229	0.528
BDI-II × Sample (Online Workers)	0.259 (0.131)	1,076.704	1.980	0.048	0.124
Sex (Male) × Sample (Online Workers)	−0.185 (0.221)	1,076.704	−0.836	0.403	0.645
Task Stage (Intermixed) × Sample (Online Workers)	−0.200 (0.171)	914	−1.171	0.242	0.528
Task Stage (Reversal) × Sample (Online Workers)	0.032 (0.171)	914	0.186	0.852	0.895
BDI-II × Sex (Male) × Task Stage (Intermixed)	−0.043 (0.230)	914	−0.187	0.851	0.895
BDI-II × Sex (Male) × Task Stage (Reversal)	0.084 (0.230)	914	0.367	0.714	0.895
BDI-II × Sex (Male) × Sample (Online Workers)	−0.062 (0.243)	1,076.704	−0.254	0.800	0.895
BDI-II × Task Stage (Intermixed) × Sample (Online Workers)*	−0.305 (0.147)	914	−2.082	0.038	0.129
BDI-II × Task Stage (Reversal) × Sample (Online Workers)	−0.161 (0.147)	914	−1.101	0.271	0.543
Sex (Male) × Task Stage (Intermixed) × Sample (Online Workers)	0.239 (0.248)	914	0.963	0.336	0.620
Sex (Male) × Task Stage (Reversal) × Sample (Online Workers)	0.179 (0.248)	914	0.719	0.472	0.667
BDI-II × Sex (Male) × Task Stage (Intermixed) × Sample (Online Workers)	0.023 (0.273)	914	0.085	0.932	0.932
BDI-II × Sex (Male) × Task Stage (Reversal) × Sample (Online Workers)	−0.156 (0.273)	914	−0.573	0.567	0.756
Random Effects	Variance (s^2^)	SD			
Participant (Intercept)	0.349	0.591			
Residual	0.596	0.772			

Standardized Coefficients (SE). BDI-II (prop. score) = depressive score on the Beck Depression Inventory-II, expressed as a proportion of total possible score. Active avoidance accuracy and BDI-II scores were z-normalized using the grand mean. p values were adjusted using the Benjamini–Hochberg false discovery rate method. Related models in Extended Data Tables 3-1 (BAI–Active), 3-2 (BDI-II-Inhibitory), and 3-3 (BAI–Inhibitory).

* *p* < 0.05.

** *p* < 0.01.

*** *p* < 0.001.

10.1523/ENEURO.0034-25.2025.t3-1 Table 3-1 Download Table 3-1, TEX file.

10.1523/ENEURO.0034-25.2025.t3-2 Table 3-2 Download Table 3-2, TEX file.

10.1523/ENEURO.0034-25.2025.t3-3 Table 3-3 Download Table 3-3, TEX file.

*Depressive symptom scores and inhibitory avoidance accuracy.* A similar linear mixed model was used to examine the relationship between depressive symptom scores (BDI-II symptom scores, *z*-normalized, grand-mean centered) and inhibitory avoidance accuracy (*z*-normalized, grand-mean centered). The model included Sex (female, male), Task Stage (intermixed, reversal), and Sample (undergraduates, online workers) as fixed effects and Participant as a random intercept. No main effects or interactions involving BDI-II were significant (Extended Data Table 3-2), suggesting that depressive symptoms were not associated with inhibitory avoidance performance using this task.

### Study 2 (reward-seeking/avoidance)

In Study 1, participants showed lower accuracy on active compared with inhibitory avoidance trials. However, it remained unclear whether this effect was driven by conflict arising from a prepotent tendency to inhibit action under threat (Bolles, 1970; Pessoa, 2009; Wendt et al., 2017) or by a preference to reduce effort expenditure due to the additional demands of effortful active responses (Hogan et al., 2020; Forys et al., 2023). To address this, Study 2 used a mixed-motivation task that assesses both active and inhibitory responses within reward-seeking and avoidance contexts in undergraduates. Here, the design manipulated the congruency between motivational context (reward-seeking vs avoidance) and instrumental response (active vs inhibitory), allowing for analysis of how motivational context shapes action tendencies. Moreover, because the ACDM framework proposes that depression is associated with altered reward-seeking and effort-related decision-making (Bishop and Gagne, 2018), we examined whether individual differences in depressive symptom scores would differentially affect behavior across motivational contexts. This design allowed for a detailed examination of both reward-seeking and avoidance behaviors, considering their active and inhibitory dimensions.

**Figure 6.**
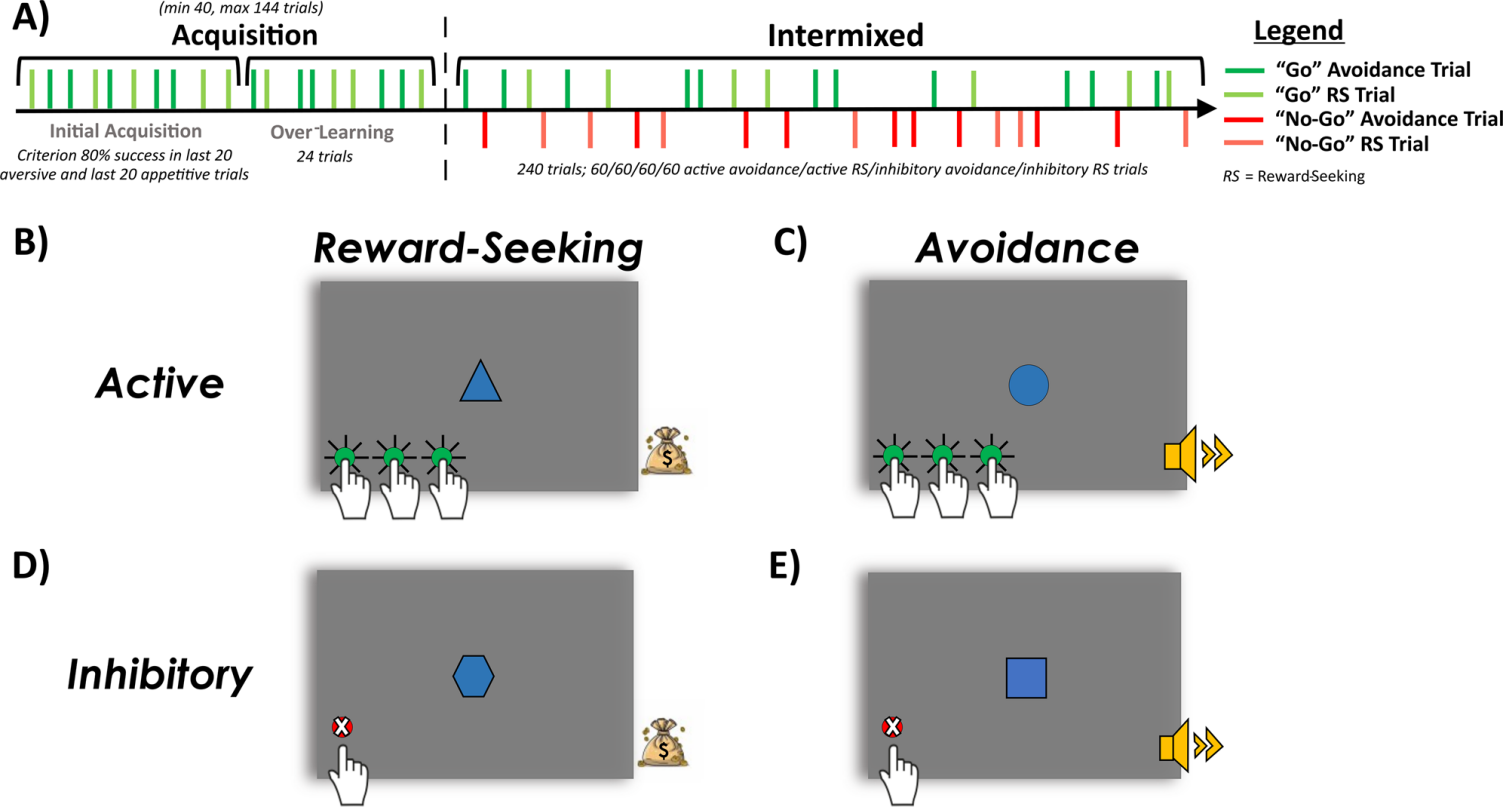
Study 2: Mixed-motivation go/no-go task. ***A***, Schematic of task stages. Acquisition and intermixed. Green lines represent active (“Go”) trials, while red lines represent inhibitory (“No-Go”) trials. Light green and light red lines indicate reward-seeking (RS) trials, while dark green and dark red lines indicate avoidance trials. The black arrow indicates time progression. During acquisition, participants were required to reach a criterion of 80% correct active reward-seeking and 80% correct active avoidance trials over a 20-trial period (i.e., initial acquisition) or complete a maximum of 144 trials. Upon reaching criterion, participants completed 24 over-learning trials, followed by an unsignaled transition into the intermixed stage. In this stage, participants flexibly alternated between active and inhibitory reward-seeking and active and inhibitory avoidance responses. ***B***, Active reward-seeking: A blue triangle signals an active reward-seeking trial. The participant makes sufficient keyboard presses (green circles) within 1,200 ms cue period and is awarded points (+5 points) toward a monetary reward, followed with a reward-signal (white border onscreen, not shown). ***C***, Active avoidance: A blue circle signals an active avoidance trial. The participant makes sufficient keyboard presses (green circles) within 1,200 ms cue period, avoiding an aversive sound (yellow speaker), followed with a safety signal (white boarder onscreen, not shown). ***D***, Inhibitory reward-seeking: A blue hexagon signals an inhibitory reward-seeking trial. The participant withholds keyboard presses for the full 1,200 ms and is awarded points toward a monetary reward, followed with a reward-signal. ***E***, Inhibitory avoidance: A blue square signals an inhibitory avoidance trial. The participant withholds keyboard presses for the full 1,200 ms, avoiding an aversive sound, followed by a safety signal. Failed reward-seeking or avoidance trials result in no points (+0 points) or the presentation of an aversive sound, respectively.

We hypothesized that task accuracy would be highest for inhibitory avoidance and active reward-seeking, as these behaviors are contextually aligned with prepotent response tendencies—inhibiting action to avoid threat and initiating action to obtain reward. Furthermore, we expected that participants with higher depressive symptom scores would exhibit reduced accuracy in active reward-seeking, consistent with predictions of diminished behavioral engagement due to effort demand overestimation and/or reward undervaluation. A graphical overview of the mixed-motivation task is provided in Figure 6.

#### Demographics

**Figure 7.**
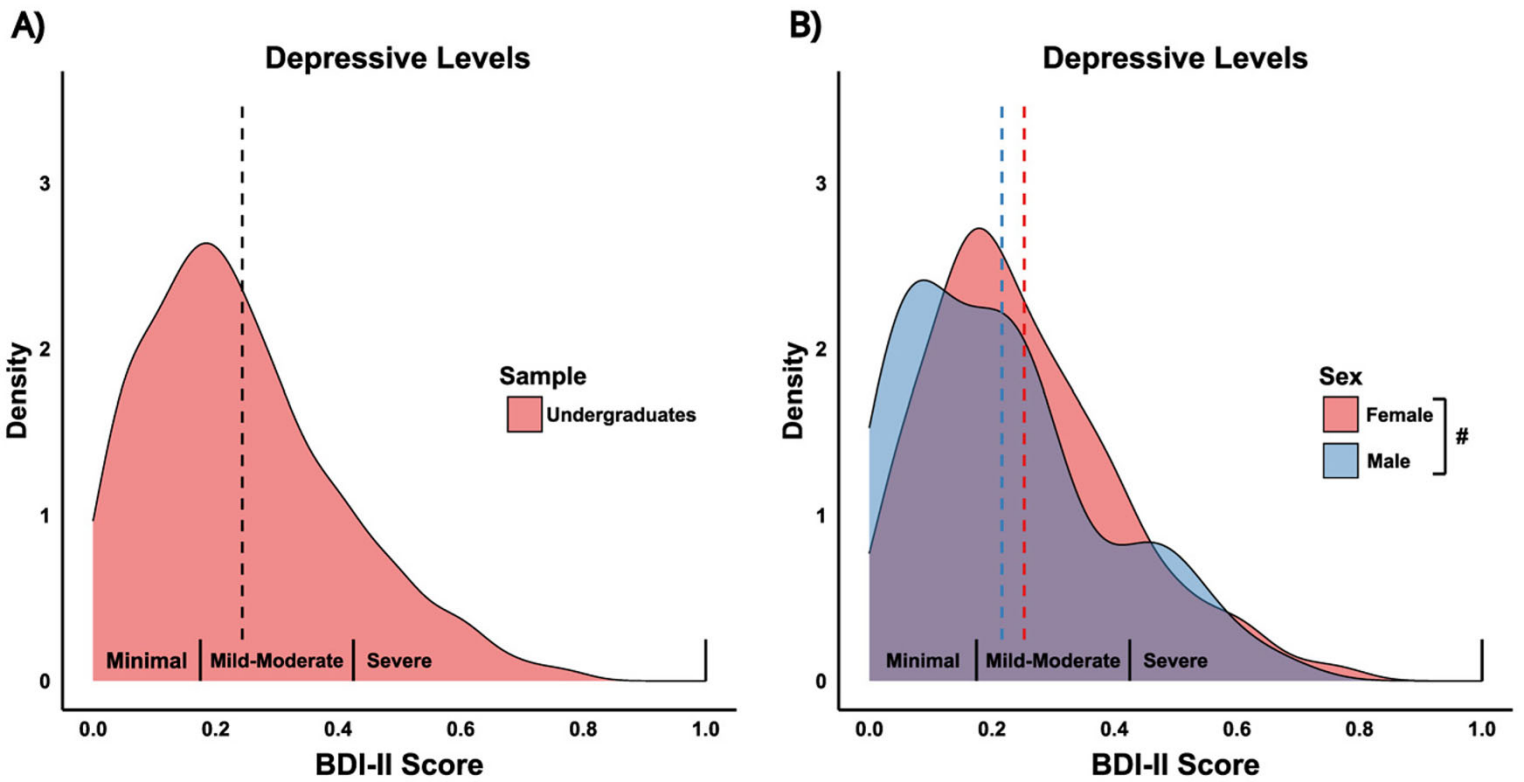
Study 2: Distribution of depressive symptom scores in undergraduates and across sexes. Density plots representing the distribution of Beck Depression Inventory-II (BDI-II) symptom scores. ***A***, Depressive symptom score distributions in full undergraduate sample (red). ***B***, Depressive symptom score distributions by sex: Female (red) and Male (blue). The *x*-axis represents proportion scores, where raw BDI-II symptom scores, ranging from 0 to 60, have been divided by the maximum possible score (60) to produce a proportion between 0 and 1. This adjustment was made because the suicide ideation question was removed for ethical consideration. The labels on the *x*-axis—Minimal (0–13), Mild-Moderate (14–28), Severe (29–63)—reflect typical ranges of raw symptom scores for ease of interpretation. Dashed vertical black line represents mean in full sample. Dash vertical-colored lines represent the mean BDI-II symptom score for each group. In panel ***B***, a marginal significant difference in depressive levels between sexes is indicated (*p* < 0.10), with females scoring higher on average than males. See Extended Data Figures 7-1 (BAI distributions) and 7-2 (Age distribution).

Females reported marginally higher levels of depressive symptoms (*F*_(1,328)_ = 3.24, *p* = 0.0729) and significantly higher levels of anxiety symptoms (*F*_(1,328)_ = 12.99, *p* < 0.001) compared with males (Fig. 7*B*; Extended Data Fig. 7-1). There was no significant age difference between sex (*F*_(1,328)_ = 0.112, *p* = 0.738).

10.1523/ENEURO.0034-25.2025.f7-1 Figure 7-1 **Study 2: Distribution of Anxiety Scores in Undergraduates and Across Sexes.** Density plots representing the distribution of Beck Anxiety Inventory (BAI) scores. **A)** Anxiety score distributions in full undergraduate sample (red). **B)** Anxiety score distributions by sex: Female (red) and Male (blue). The x-axis represents proportion scores, where raw BAI scores, ranging from 0-63, have been divided by the maximum possible score (63) to produce a proportion between 0 and 1. This adjustment was made for comparability between the BDI-II and BAI scales. The labels on the x-axis -- Minimal (0-7), Mild-Moderate (8-25), Severe (26-63) -- reflect typical ranges of raw scores for ease of interpretation. Dashed vertical black line represents mean in full sample. Dashed vertical-coloured lines represent the mean BAI score for each sex. In panel B, a significant difference in anxiety levels between sexes is indicated (*p* < .001), with females scoring higher on average than males. Download Figure 7-1, TIF file.

10.1523/ENEURO.0034-25.2025.f7-2 Figure 7-2 **Study 2: Age Distribution in Undergraduates.** Density plot representing the distribution of ages for undergraduates (red). Dashed vertical black line represent the mean age. Download Figure 7-2, TIF file.

#### Mixed-motivation go/no-go task

##### Acquisition

**Figure 8.**
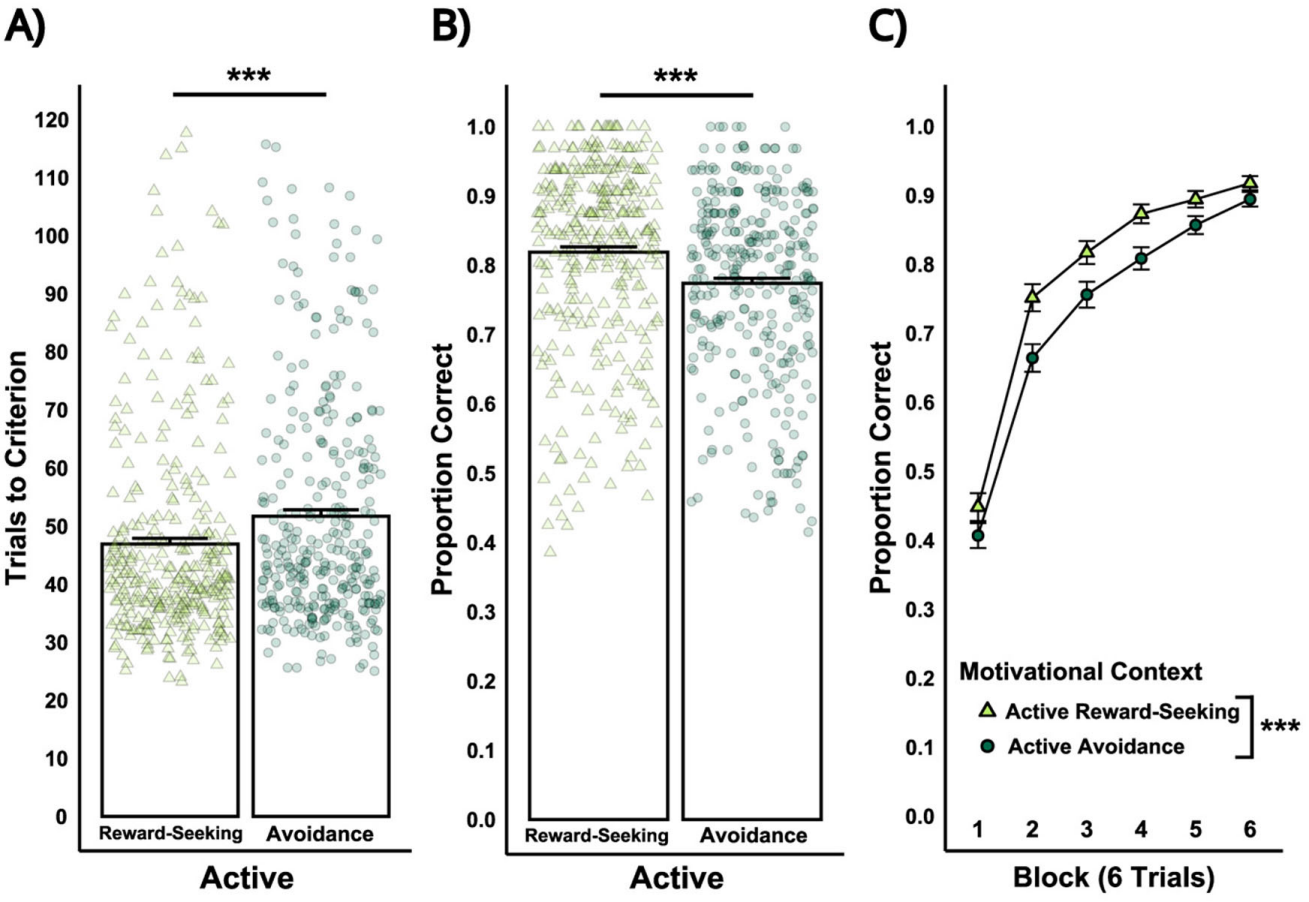
Study 2: Performance comparison between active reward-seeking and active avoidance during the acquisition stage. ***A***, Number of trials required to reach criterion for all participants in the mixed-motivation task. Individual data points (light green triangles for Active Reward-Seeking; dark green circles for Active Avoidance) represent the number of trials each participant required to reach the criterion of 80% correct responses within 20-trial period during acquisition. ***B***, Overall accuracy for active reward-seeking and active avoidance in the acquisition stage. Individual data points (light green triangles for Active Reward-Seeking; dark green circles for Active Avoidance) represent the proportion correct for each participant. Proportion correct reflects the ratio of successful active responses relative to the total number of trials within each motivation type. ***C***, Accuracy for active reward-seeking and active avoidance across blocks of six trials, with triangles and circles representing mean performance for reward-seeking and avoidance trials, respectively. Significant differences between active reward-seeking and active avoidance during the acquisition stage are indicated (*p* < 0.001).

Participants successfully learned both active reward-seeking and avoidance responses, as indicated by the number of trials to criterion (reward-seeking: *M* = 46.94, SD = 18.09; avoidance: *M* = 51.72, SD = 19.90). A one-way within-subjects ANOVA revealed a significant effect of Motivational Context on the number of trials to criterion, with more trials needed to acquire active avoidance than reward-seeking (*F*_(1,329)_ = 30.96, *p* < 0.001; Fig. 8*A*). To test whether depressive symptoms predicted trials to criterion, we fit a linear mixed model including BDI-II scores, Sex, and Motivational Context. No main or interaction effect of BDI-II was observed. Motivational context significantly affected acquisition accuracy, with lower proportion correct on active avoidance (*M* = 0.774, SD = 0.135) compared with active reward-seeking (*M* = 0.819, SD = 0.137; *F*_(1,329)_ = 52.59, *p* < 0.001; Fig. 8*B*). To assess how this difference varied over time, we analyzed accuracy across the first six trial blocks (where all participants had data). Accuracy improved across blocks (main effect of block) and remained higher for reward-seeking trials compared with avoidance trials (main effect of Motivational Context; Table 4). While this difference persisted across blocks 1–5 (*t*'s_(1629)_ > 2.56, *p*'s < 0.01), it converged by block 6 (*t*_(1,629)_ = 1.59, *p* = 0.11; Fig. 8*C*), suggesting slower acquisition for active avoidance than reward-seeking.

**Table 4.** Study 2—2 × 6 within-subjects ANOVA for active response accuracy for acquisition task stage by block

Proportion Correct ∼ Motivational Context × Block + Error (Participant/(Motivational Context × Block))						
Effect	df	Sum Sq	Mean Sq	*F* value	*p* value	*η*^2^ (partial)
Within-Subjects						
Motivational Context***	1	2.73	2.73	38.20	<0.001	0.10
Residuals (Motivational Context)	329	23.53	0.07			
Block***	5	102.30	20.47	276.70	<0.001	0.46
Residuals (Block)	1,645	121.70	0.07			
Motivational Context × Block**	5	0.44	0.09	3.11	<0.01	0.01
Residuals (Motivational Context × Block)	1,645	46.07	0.03			
Participant						
Residuals	329	147.90	0.45			

** *p* < 0.01.

*** *p* < 0.001.

##### Intermixed

**Figure 9.**
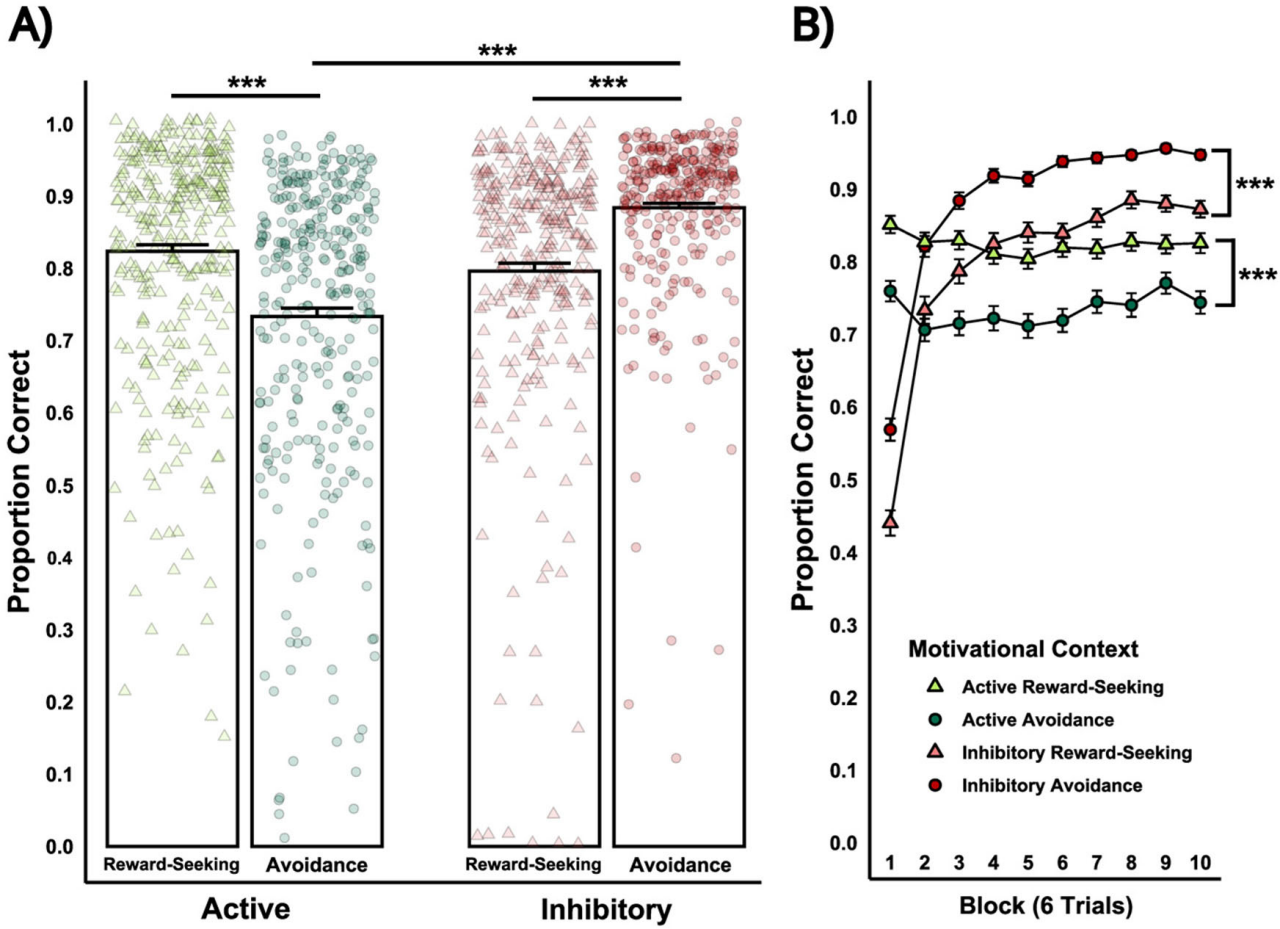
Study 2: Performance comparison between reward-seeking and avoidance for active and inhibitory responses during the intermixed stage. ***A***, Overall accuracy for active/inhibitory reward-seeking and avoidance in the intermixed stage. Individual data points (light green triangles for Active Reward-Seeking; light red triangles for Inhibitory Reward-Seeking, dark green circles for Active Avoidance, dark red circles for Inhibitory Avoidance) represent the proportion correct for each participant. Proportion correct reflects the ratio of successful responses relative to the total number of trials within each trial type. ***B***, Accuracy for active/inhibitory reward-seeking and avoidance across blocks of six trials during the intermixed task stage. Shapes represent mean performance for each trial type across 10 blocks. Significant differences between (1) active reward-seeking and active avoidance, (2) inhibitory reward-seeking and inhibitory avoidance, and (3) active and inhibitory avoidance are indicated (*p* < 0.001).

The intermixed stage assessed participants’ ability to flexibly select actions or inhibit responses based on motivational contexts (i.e., reward-seeking vs avoidance). A 2 × 2 within-subjects ANOVA (Motivational Context × Response Type) revealed a significant main effect of Response Type and a significant interaction but no main effect of Motivational Context (Table 5). Follow-up analysis revealed higher accuracy for active reward-seeking than active avoidance (*t*_(658)_ = 9.92, *p* < 0.0001) and higher accuracy for inhibitory avoidance compared with inhibitory reward-seeking (*t*_(658)_ = 9.60, *p* < 0.0001). In the avoidance context, inhibitory responses were more accurate compared with active responses (*t*_(658)_ = 10.73, *p* < 0.0001), consistent with Study 1. There were no accuracy differences in Response Type in the reward-seeking context (*t*_(498)_ = 1.95, *p* = 0.21; Fig. 9).

**Table 5.** Study 2—2 × 2 within-subjects ANOVA for active and inhibitory response accuracy for intermixed task stage

Proportion Correct ∼ Motivational Context × Response Type + Error (Participant/(Motivational Context × Response Type))						
Effect	df	Sum Sq	Mean Sq	*F* value	*p* value	*η*^2^ (partial)
Within-Subjects						
Motivational Context	1	0.001	0.001	0.05	0.821	0.00
Residuals (Motivational Context)	329	4.398	0.013			
Response Type***	1	1.254	1.254	24.58	<0.001	0.08
Residuals (Response Type)	329	16.786	0.051			
Motivational Context × Response Type***	1	2.615	2.615	185.60	<0.001	0.36
Residuals (Motivational Context × Response Type)	329	4.635	0.014			
Participant						
Residuals	329	14.710	0.045			

*** *p* < 0.001.

#### Depressive symptom scores and active response accuracy

We used a linear mixed model to examine whether depressive symptoms (BDI-II, *z*-normalized, grand-mean centered) predicted accuracy on active trials (also *z*-normalized). Fixed effects included Sex (female, male), Task Stage (acquisition, intermixed), and Motivational Context (reward-seeking, avoidance), with Participant as a random intercept. This structure matched Study 1, with motivational context added. Motivational Context significantly influenced accuracy, with lower performance on avoidance trials (avoidance; *β* =−0.307, SE = 0.075, *t*_(978)_ = −4.11, *p* < 0.001). However, BDI-II symptom scores were not significantly associated with active accuracy (*β* = 0.035, SE = 0.063, *t*_(1,050.90)_ = 0.56, *p* = 0.85), nor did they interact with Motivational Context (avoidance; *β* = 0.038, SE = 0.076, *t*_(978)_ = 0.496, *p* = 0.85). Thus, although avoidance reduced active accuracy this effect was not associated with depressive symptom scores. Full model results can be found at https://osf.io/5sepm/.

To evaluate whether observed effects were sensitive to reference level selection, we conducted an exploratory series of eight linear mixed models, systematically varying the reference levels for Sex, Task Stage, and Motivational Context. This resulted in 128 tested effects (16 per model, including main effects and interactions), and *p* values were adjusted using Benjamini–Hochberg FDR across all 128 effects. While no significant effects of BDI-II emerged in the initial model, exploratory analyses identified a significant BDI-II × Sex interaction (*β* = −0.365, SE = 0.121, *t*_(1,050.90)_ = −3.02, *p*_adjusted_ = 0.026), specifically in the avoidance context during the intermixed stage. This exploratory finding suggests the possibility that sex differences in the relationship between depressive symptoms and instrumental behavior may emerge when active responses are well-learned and inhibitory demands are newly introduced—potentially reflecting sex-specific dynamics in threat processing during later phases of learning. Full model results can be found at https://osf.io/5sepm/.

#### Depressive symptom scores and inhibitory response accuracy

A similar linear mixed model was used to examine whether depressive symptom scores (BDI-II, *z*-normalized) predicted accuracy on inhibitory trials. Fixed effects include Sex (female, male) and Motivational Context (reward-seeking, avoidance) and Participant as a random intercept. Motivational Context significantly affected inhibitory accuracy, with higher performance in the avoidance context (avoidance; *β* = 0.570, SE = 0.064, *t*_(326)_ = 8.95, *p* < 0.001). However, BDI-II symptom scores were not significantly associated with inhibitory accuracy (*β* = −0.074, SE = 0.063, *t*_(534.47)_ = −1.18, *p* = 0.28), nor was there a significant interact with BDI-II and Motivational Context (avoidance; *β* = 0.030, SE = 0.065, *t*_(326)_ = 0.47, *p* = 0.64). Thus, although participants showed lower accuracy for inhibitory reward-seeking trials compared with avoidance trials, this pattern was not associated with depressive symptom severity.

## Discussion

In this study we report that higher depressive scores are associated with a reduced capacity to learn active avoidance behaviors, while no relationship was observed with inhibitory avoidance. Specifically, Study 1 extended rodent research on active and inhibitory avoidance to a human nonclinical sample, revealing that higher depressive symptoms predicted lower accuracy during the acquisition phase of active avoidance (Fig. 5) and a greater number of trials required to reach criterion performance (Fig. 4*B*). In contrast, depressive symptom scores were not related to performance on inhibitory avoidance trials. Additionally, within-subjects analyses indicated that overall, inhibitory avoidance was performed more readily compared with active avoidance, as indicated by higher accuracy during the intermixed and reversal stages (Fig. 3*B*,*C*). Altogether, these findings highlight a selective impairment in active avoidance learning associated with depressive symptoms and underscore the importance of considering how this relationship may vary across different learning phases. This dynamic pattern warrants further investigation into the underlying cognitive and neural processes that constrain avoidance behavior in depression.

Our findings in Study 1 partially support the predictions of the ACDM framework, which posit that depression is associated with a greater tendency toward inaction in avoidance contexts (Bishop and Gagne, 2018). Using this framework, the decision to act is calculated as the difference between the product of estimated outcome value and probability and the estimated cost of deploying effort to obtain a desired outcome. In depression, inaction may arise from overestimating effort costs, undervaluing outcomes, or underestimating outcome probability. Notably, because our task used a deterministic reinforcement schedule, outcome uncertainty is unlikely to account for the observed deficit. If overestimation of effort costs were solely responsible for these deficits, one would expect consistent active avoidance impairments across all task stages. However, since effort demands remained constant (i.e., the number of button presses required to obtain the desired outcome) and inaction was most pronounced during the acquisition phase—when fatigue-related effort costs were likely minimal—alternative explanations must be considered. Another possibility is that individuals with elevated levels of depressive symptoms became increasingly sensitive to the aversive outcome over time—effectively overvaluing the punishment and potentially overriding initial biases against deploying effort. Consistent with this interpretation, several studies have demonstrated that individuals with depression show increased sensitivity to negative feedback (Elliott et al., 1997; Eshel and Roiser, 2010), particularly in probabilistic reversal learning tasks, where they are more likely to switch following misleading negative feedback (Murphy et al., 2003; Taylor Tavares et al., 2008). However, other work suggests that the negativity bias in depression may not reflect punishment hypersensitivity per se, but rather blunted responsiveness to reward, resulting in a relative overweighting of negative outcomes (Robinson et al., 2012). Still other studies have reported reduced sensitivity to both reward and punishment in depressed individuals (Mukherjee et al., 2020). This heterogeneity likely reflects differences in task structure (i.e., deterministic vs probabilistic reinforcement), cognitive control demands (i.e., attending to and memorizing cue–response associations), and sample characteristics such as comorbid anxiety, sex, IQ, and medication status. Regardless, our findings suggest that the relationship between depressive symptoms and active avoidance is dynamic, with experience-dependent shifts across phases of avoidance.

Clarifying how the neural circuits regulating active avoidance are dynamically engaged over time may offer critical insight into motivational dysfunction in depression. Evidence from both human and animal studies highlights the role of species-specific defensive reactions (SSDRs), where freezing is a prepotent response in aversive contexts (Bolles, 1970; Fanselow, 1994; LeDoux et al., 2017). For successful active avoidance, both humans and rodents must overcome these prepotent defensive responses to engage in instrumental, goal-directed action. From a neural circuitry perspective, considerable progress has been made in understanding the mechanisms underlying the acquisition of active avoidance (LeDoux et al., 2017; Cain, 2019). Early in avoidance training, SSDRs are largely driven by amygdala circuits that promote behavioral suppression. With repeated training, however, ventromedial prefrontal systems (homologs of infralimbic cortex, Area 25 of anterior cingulate) increasingly suppress amygdala activity to reduce freezing and facilitate goal-directed avoidance responses (Moscarello and LeDoux, 2013). These dynamics suggest that individuals with elevated depressive symptoms may exhibit difficulty in suppressing prepotent defensive responses during early learning—potentially due to dysfunction in cortico-limbic-striatal circuits that support the shift from reactive to goal-directed control. This provides a plausible neurobiological mechanism for the symptom-related impairments in active avoidance observed during the acquisition phase, while performance at later stages remains unaffected.

Moving to research in humans, the dual competition model (Pessoa, 2009) proposes the effects of emotionally salient stimuli on task performance depends both on the level of arousal evoked by a stimulus and on whether the stimulus aligns with or opposes the action tendency evoked by the stimulus. Prepotent behavioral responses to avoid punishment and approach reward, mediated in part by prefrontal regions, have been reliably observed in human neuroimaging studies (Guitart-Masip et al., 2012; Asci et al., 2019). In depression, disruptions in top-down regulatory control have been linked to reduced activity in dorsolateral and dorsomedial prefrontal cortex (dlPFC, dmPFC) and rostral ACC (rACC), along with elevated and sustained amygdala activity in response to negative feedback or emotional salient stimuli (Siegle et al., 2007; Fales et al., 2008; Taylor Tavares et al., 2008). This pattern may indicate that emotionally salient cues disproportionately influence behavior due to weakened regulatory input from cognitive control systems. As a result, the capacity to override prepotent defensive responses—particularly during early stages of active avoidance learning—may be compromised in depression. Recent work further supports this interpretation, showing that reductions in GABA within the rACC were associated with decreased functional connectivity across cortico-striatal-limbic circuits in females with MDD (Ironside et al., 2021)—a finding especially relevant given our predominantly female sample.

### Avoidance mechanisms in depression and related disorders

To contextualize our findings, it is important to position them within the broader literature on avoidance across psychiatric disorders, highlighting key conceptual differences and points of convergence. For instance, many studies define avoidance as the decreased selection of high-loss options in probabilistic selection tasks—a definition that differs meaningfully from the framework used here but useful for understanding sensitivity to reward and negative feedback. Chase et al. (2010) used a probabilistic selection task to examine feedback learning in individuals with MDD and found reduced learning rates for both positive and negative feedback during training, particularly among individuals with higher anhedonia. This suggests blunted reinforcement learning rather than a valence-specific bias such as altered sensitivity to negative feedback. Nonetheless, this profile is consistent with our observed impairment in active avoidance acquisition, despite differences in task design.

Mukherjee et al. (2020) extended this work using probabilistic reversal learning and found that MDD patients—most of whom were medicated—selected fewer rich options following reversals and exhibited reduced win-stay behavior (i.e., less likely to repeat a rewarded choice), but no difference in lose-shift behavior (i.e., switching after punishment). If depression involved heightened punishment sensitivity, an increase in lose-shift behavior would be expected. The absence of this effect supports the idea of diminished reward sensitivity rather than increased responsiveness to punishment. This interpretation aligns with the possibility that symptom-related impairments in active avoidance reflect deficits in safety learning rather than heightened punishment sensitivity that interacts with effort-related biases. Safety learning—the process of learning about cues that predict the absence of threat (Laing et al., 2025)—has been shown to promote instrumental avoidance learning in animals and humans (Fernando et al., 2014; Fisher and Urcelay, 2024). Impaired learning of safety signals may contribute to reduced active avoidance performance, even in aversively motivated contexts. Given that safety learning is supported by amygdala and vmPFC circuitry (Kong et al., 2014), this may offer a more parsimonious explanation for acquisition-specific effects than models emphasizing the accumulation of punishment sensitivity and effort-related bias.

Neuromodulator systems may further complicate interpretation, as both serotonin and dopamine are implicated in punishment and reward learning. SSRIs, commonly prescribed in MDD, are known to blunt negative feedback sensitivity (Herzallah et al., 2013). Supporting this, low doses of the antidepressant citalopram—which attenuate serotonin signaling—increase lose-shift behavior and sensitivity to punishment in both rodents and humans (Chamberlain et al., 2006; Bari et al., 2010). However, findings from obsessive compulsive disorder (OCD) populations highlight more nuanced effects of serotonergic modulation: Endrass et al. (2011) found greater sensitivity to negative feedback in medicated OCD patients with elevated depressive symptoms, but only after initial learning—consistent with the idea that punishment sensitivity may build with experience. In contrast, Palminteri et al. (2012) reported no valence-specific effects of medication status in OCD patients using a task previously linking dopamine to punishment learning (Palminteri et al., 2009).

Motivational impairments similar to those in depression are also evident in schizophrenia, particularly in relation to altered dopamine signaling. In unmedicated patients, Reinen et al. (2016) found blunted prediction error BOLD signals in the striatum and mPFC for rewards, but intact response to punishment, suggesting D2 tone may selectively dampen reward while keeping punishment signaling intact. Similarly, Waltz et al. (2018) reported reduced differential activation to gain versus loss-avoidance in vmPFC, ACC, and ventral striatum (VS), with diminished activation in VS associated with higher negative symptom scores. Sex differences in dopaminergic responses to loss versus gain have also been observed. Using PET during the monetary incentive delay task, Hahn et al. (2021) found females exhibited heightened VS dopaminergic responses to punishment relative to gain. Together, these findings suggest that disrupted valuation and motivational processes, linked to both dopamine and serotonin signaling, may reflect cortico-striatal-limbic dysfunction as a transdiagnostic mechanism across conditions like OCD, schizophrenia, and depression.

Overall, these studies underscore the dynamic nature of avoidance, which may shift with experience (i.e., acquisition, expression, habit) and neuromodulatory state. If punishment sensitivity increases with experience, it may eventually override early inaction driven by effort-related biases. Alternatively, if deficits are more prominent for reward-related signals, disrupted safety learning may play a greater role. Future computational modeling that integrates effort costs, punishment and reward sensitivity, and safety learning mechanisms will be critical for disentangling these processes and clarifying how depressive symptoms influence active avoidance behavior.

### Motivational context influences accuracy of instrumental actions

Study 2 examined whether poorer active avoidance performance reflect a general bias toward effort minimization or context-specific effects by assessing active and inhibitory responses across both reward-seeking and avoidance contexts. While depressive symptom scores were not significantly related to performance, robust within-subjects effects emerged. Participants performed more accurately on trials aligned with their prepotent tendencies—active reward-seeking and inhibitory avoidance—consistent with prior research demonstrating approach biases for reward and withdrawal biases for punishment (Crockett et al., 2009; Guitart-Masip et al., 2012; Mkrtchian et al., 2017; Asci et al., 2019).

Prefrontal regions such as the anterior prefrontal cortex (aPFC) and orbitofrontal (OFC) are implicated in overcoming these motivational-action conflicts (Roelofs et al., 2009; Volman et al., 2011). If the poorer performance for active versus inhibitory avoidance observed in Study 1 were driven by a general preference to minimize effort, we would expect a similar pattern for active reward-seeking in Study 2. However, this pattern did not emerge, suggesting that effort bias alone does not account for these findings.

Parallel findings in humans and animals suggest that newly learned discriminative cues can differentially influence instrumental behavior depending on whether the context is appetitive or aversive (Talmi et al., 2008; Geurts et al., 2013; Mkrtchian et al., 2017; Nord et al., 2018; Campese, 2021). Consistent with this, participants in the current study more readily inhibited responses in avoidance context than in reward-seeking contexts. Remarkably, rats display similar patterns, with greater accuracy for active responses during reward seeking and greater inhibitory accuracy during avoidance (Dalton et al., 2025).

Although these performance differences could be attributed to differences in the motivational value of the reward and the punishment outcomes, this explanation is unlikely. No significant main effect of motivational context was found during the intermixed task stage. Specifically, the average accuracy for reward-seeking and avoidance trials (irrespective of response type) did not differ. This suggests that the outcomes were equally motivating overall and not biased toward one context. Instead, the observed interaction is more consistent with the context-dependent effect of prepotent response tendencies influencing instrumental actions.

While depression is typically associated with reduced reward-seeking, the ACDM framework predicts a broader bias toward inaction across both reward-seeking and avoidance contexts, driven by overestimation of effort costs. In Study 1, higher depressive symptom scores were associated with reduced active avoidance performance—consistent with this framework. However, contrary to our hypotheses, depressive symptoms were not significantly associated with active or inhibitory response accuracy in either motivational context.

Several key differences may account for these null findings. First, the mixed-motivation task employed an interleaved design, requiring participants to frequently switch between responding to appetitive and aversive stimuli, rather than engaging with each in distinct blocks. This design placed avoidance trials within a broader reward-rich context, attenuating depression-related impairments in active avoidance—potentially due to the prepotent tendency to approach reward. This interpretation aligns with findings from approach-avoidance conflict paradigms—where conditions involving potential reward despite the risk of punishment are more likely to elicit active approach behavior than avoidance-only conditions (Aupperle et al., 2011). Second, the four-condition task structure (active vs inhibitory and reward-seeking vs avoidance) likely imposed greater working memory demands, which have been implicated in reward/punishment learning (Van Der Schaaf et al., 2014). These cognitive demands may have masked the influence of depressive symptoms on performance. Future studies may consider controlling for cognitive load to better isolate symptom-specific effects. Finally, Study 1 took place during the height of the COVID-19 pandemic, a contextual factor that may have influenced affective states and task engagement in ways that were not present during Study 2.

Although no association between depressive symptoms were found in Study 2, the robust within-subjects effects suggest this task may be useful for assessing motivated behavior in other psychiatric populations. For example, research on substance use disorder emphasizes the strong motivational salience of reward and punishment related cues, particularly those associated with drug use.

### Conclusion

Although depression is often linked with reward-processing deficits like anhedonia (Treadway and Zald, 2011; Treadway et al., 2012), our findings reveal a novel link between symptom severity and impaired active avoidance learning in aversive contexts. A key limitation is whether these results generalize to clinical populations. Depression is increasingly understood as a dimensional condition, with clinical diagnoses reflecting the more severe end of a broader symptom spectrum and avoidance impairments representing a potential transdiagnostic feature (Eaton et al., 2023). Our sample included a wide range of depressive symptom scores, with many participants self-reporting prior diagnoses or scoring above clinical cutoffs, supporting the relevance of our findings to clinical populations. Furthermore, this dimensional approach may offer a more nuanced understanding of symptom-related effects on avoidance behavior and extend to other psychiatric conditions, such as OCD and schizophrenia, that share overlapping motivational and affective features with depression.

Another limitation of our study was the higher than expected exclusion rates, which warrant a closer examination of their potential impact on results. Both tasks were designed as reinforcement-based learning paradigms with minimal instructions, no explicit information on cue–response contingencies, and no practice trials. While this design enhances translational relevance, it likely contributed to the variability in participants’ ability to acquire the task contingencies. Although higher exclusion rates were anticipated, the rates observed—39.37% in Study 1 and 57.20% in Study 2—exceeded expectations and were primarily due to failure to meet behavioral performance criteria. However, when considering only exclusions related to questionnaire failures or task noncompletion, rates were consistent with typical online studies (Study 1: 19.17%, Study 2: 19.20%; Suzuki et al., 2021). To assess potential bias, we compared included participants to those who passed attention checks but were later excluded for other reasons (see https://osf.io/5sepm/). In Study 1, excluded participants had significantly higher BAI scores, but not BDI-II scores. While the ACDM framework does not explicitly make predictions about how depressive and anxiety symptoms interact, it implies that their effects may counterbalance one another. In the context of our study, anxiety symptoms could offset the depressive impairments in active avoidance by promoting increased avoidance effort. This antagonistic dynamic suggests that higher exclusion rates may have reduced confounding influences rather than introduce bias. Importantly, BDI-II scores did not differ between included and excluded groups, preserving the validity of our primary analyses. Sex differences in exclusion rates were also observed, with a higher proportion of females excluded. However, both final samples remained predominantly female—the group in which we observed our strongest effects—suggesting any bias would likely underestimate, rather than inflate our findings. For these reasons, we believe the interpretation and relevance of our findings are still valid. However, future adaptations of this task may benefit from optimizing the trade-off between ecological validity and participant retention.

Across two studies, we sought to extend rodent research to investigate patterns of active and inhibitory avoidance and reward-seeking in a nonclinical sample varying in depressive symptoms. By integrating self-report and behavioral measures, we aimed to strengthen translational links between preclinical models and depressive symptom severity in humans. Our findings highlight the value of transdiagnostic approaches in a community sample for bridging bench and clinic in understanding psychiatric disorders. Results demonstrate an important link between depressive symptoms and reduced efficacy at learning to override a prepotent response to inhibit action to avoid unpleasant events. Future work should test whether these effects replicate in clinically diagnosed MDD populations, use computational models to probe underlying mechanisms, and apply neuroimaging to evaluate cross-species convergence in neural circuitry.

## Synthesis

Reviewing Editor: Ifat Levy, Yale School of Medicine Decisions are customarily a result of the Reviewing Editor and the peer reviewers coming together and discussing their recommendations until a consensus is reached. When revisions are invited, a fact-based synthesis statement explaining their decision and outlining what is needed to prepare a revision will be listed below. The following reviewer(s) agreed to reveal their identity: Ziv Ben-Zion.

The present study examined the relationship between depressive symptoms and active/inhibitory avoidance behavior in general populations. The authors demonstrated that depressive symptoms (BDI-II scores) are negatively associated with the accuracy of active avoidance in the avoidance Go/NoGo task, particularly among younger participants. This relationship did not emerge in the mixed version of the task (i.e., a combination of reward-seeking and avoidance). Given these findings, the authors argue that their work may help bridge the gap between preclinical animal research and clinical studies.

This is an interesting paper, which may provide new insights into the relationship between depressive symptoms and avoidance learning using a reverse-translated avoidance task from rodent models. Reviewers identified, however, unclear aspects of the conceptual framework, as well as the methods and results, which should be addressed.

- The authors should provide more background, to position their study in the context of prior research. They should be clear on whether and how the work is conceptually novel. If this is a replication, this is totally fine, but should be explicitly said.

- The authors should discuss earlier research on psychiatric symptoms and avoidance behavior using simple decision-making tasks applicable to rodent models (for example: https://pubmed.ncbi.nlm.nih.gov/22420038/; https://pubmed.ncbi.nlm.nih.gov/19607754/; https://pubmed.ncbi.nlm.nih.gov/33001663/; https://pubmed.ncbi.nlm.nih.gov/22325972/; https://pubmed.ncbi.nlm.nih.gov/21284070/; https://pubmed.ncbi.nlm.nih.gov/28343697/; https://pubmed.ncbi.nlm.nih.gov/29486865/; https://pubmed.ncbi.nlm.nih.gov/27105903/; and https://pubmed.ncbi.nlm.nih.gov/34151477/).

The authors should explain how their findings relate to these previous studies.

- The authors describe the brain regions identified in animal research as related to active and inhibitory avoidance. However, it is unclear how these findings translate to humans. The statement that "Neural activation of these regions is found in humans performing avoidance tasks" is too general-are distinct brain regions implicated in active vs. inhibitory avoidance in humans? Similarly, the claim that "depression has been linked to structural or atypical patterns of activation in homologous brain regions in humans" lacks specificity. Could the authors provide more precise evidence from human neuroimaging studies to clarify these points?

- The authors clearly articulate their research objective and hypothesis, but some aspects could be clarified. First, why was a non-clinical sample chosen instead of a clinical one? How might this impact the generalizability of findings to clinical depression? Second, the hypothesis states that depressive symptoms will impair active avoidance, but do the authors expect a similar or different effect for inhibitory avoidance? Clarifying these points would strengthen the rationale for the study.

- The authors conducted numerous statistical tests as part of their main analyses. For instance, according to the description in lines 269-274, the main findings involved the effects of BDI-II and the interaction of Sex and Sample on active and inhibitory avoidance accuracies, amounting to more than 20 tests. This raises a concern about the robustness of the results - p-values should be properly corrected for multiple comparisons.

- In the mixed-effect models, why was Age omitted (i.e., replaced by Sample)? Ideally, a single model would include all relevant variables. Furthermore, it is unclear why the authors included only a random intercept rather than also considering random slopes (e.g., for task stages), which could vary across participants. Were the variables in these models z-normalized?

- The negative association between BDI-II scores and active avoidance accuracy emerged in the avoidance task but not in the mixed task. Could the authors speculate about the meaning of this discrepancy? Does it suggest that the relationship is highly context-dependent?

- In Study 1, 302 of 767 participants were excluded, and in Study 2, 439 of 769 were excluded. Such high exclusion rates - over half of each sample - are unusual. Even in online experiments on crowdsourcing services, the exclusion rate typically does not exceed 30%. It would be informative to detail what attention checks were employed and how many participants failed each criterion. This issue also warrants discussion in the Discussion section.

- Do the authors have any data regarding participants' general intelligence (e.g., educational background or IQ)? Differences between undergraduate and online participants may be attributable to such factors.

- Mental disorders frequently co-occur - in particular, depression often co-occurs with anxiety. Thus, it is possible that the BDI-II scores were influenced by symptoms other than depression. Commenting on this possibility, and on how the ACDM framework handles this would be helpful.

- Was the aversive sound truly aversive? Is there any data to support this claim?

- please provide more detail on the effort and volume calibration procedures.

- The methods and results sections are very long. Please try to streamline descriptions of procedures, statistical methods, and secondary analyses where possible.
