# Supplementary figures and images for: Depression Levels Are Associated with Reduced Capacity to Learn to Actively Avoid Aversive Events in Young Adults
Source: eNeuro. 2025 Sep 9;12(9):ENEURO.0034-25.2025. doi: 10.1523/ENEURO.0034-25.2025 (PMC12439752; doi:10.1523/ENEURO.0034-25.2025)

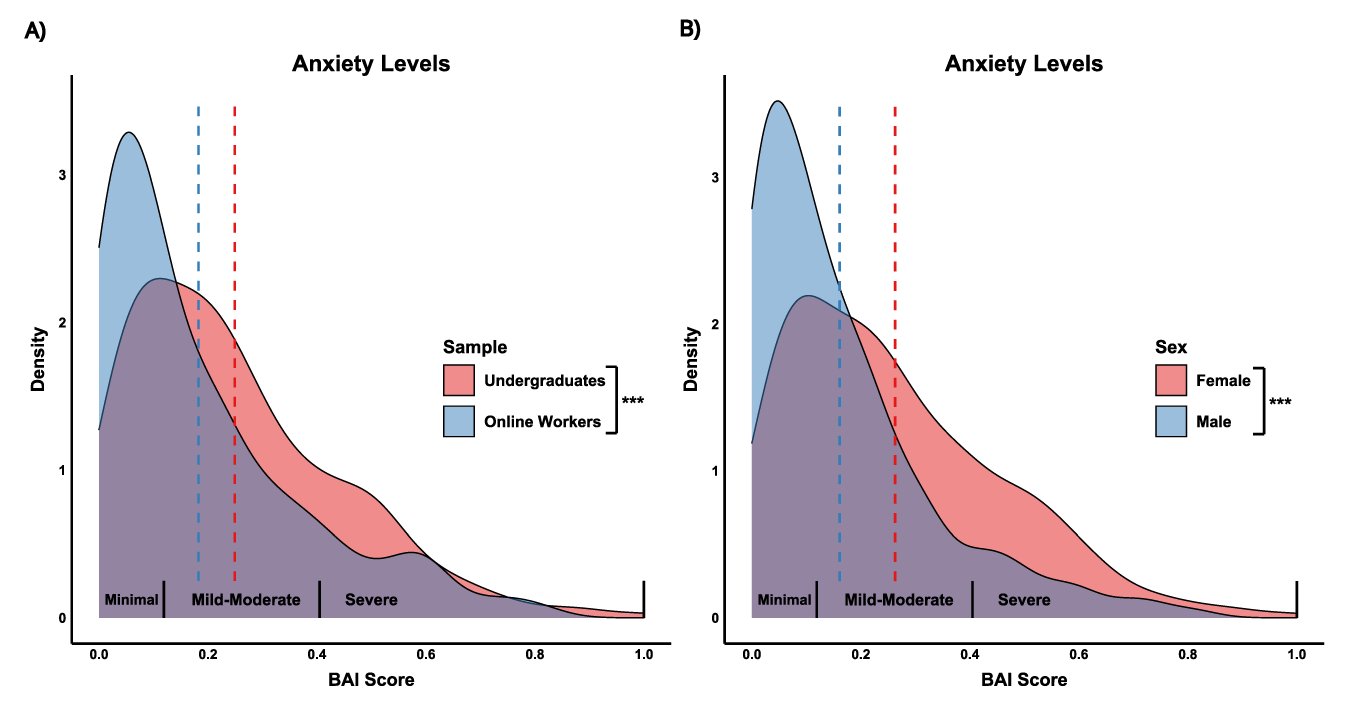

Supplement: Figure 2-1 — Study 1: Distribution of Anxiety Scores Across Samples and Sexes. Density plots representing the distribution of Beck Anxiety Inventory (BAI) scores. A) Anxiety score distributions by sample: Undergraduates (red) and Online Workers (blue). B) Anxiety score distributions by sex: Female (red) and Male (blue). The x-axis represents proportion scores, where raw BAI scores, ranging from 0-63, have been divided by the maximum possible score (63) to produce a proportion between 0 and 1. This adjustment was made for comparability between the BDI-II and BAI scales. The labels on the x-axis -- Minimal (0-7), Mild-Moderate (8-25), Severe (26-63) -- reflect typical ranges of raw scores for ease of interpretation. Dashed vertical lines represent the mean BAI score for each group. In panel A, a significant difference in anxiety levels between sample groups is indicated (p < .001), with undergraduates scoring higher on average than online workers. In panel B, a significant difference in anxiety levels between sexes is indicated (p < .001), with females scoring higher on average than males. Download Figure 2-1, TIF file. [file eneuro-12-ENEURO.0034-25.2025-s006.tif]

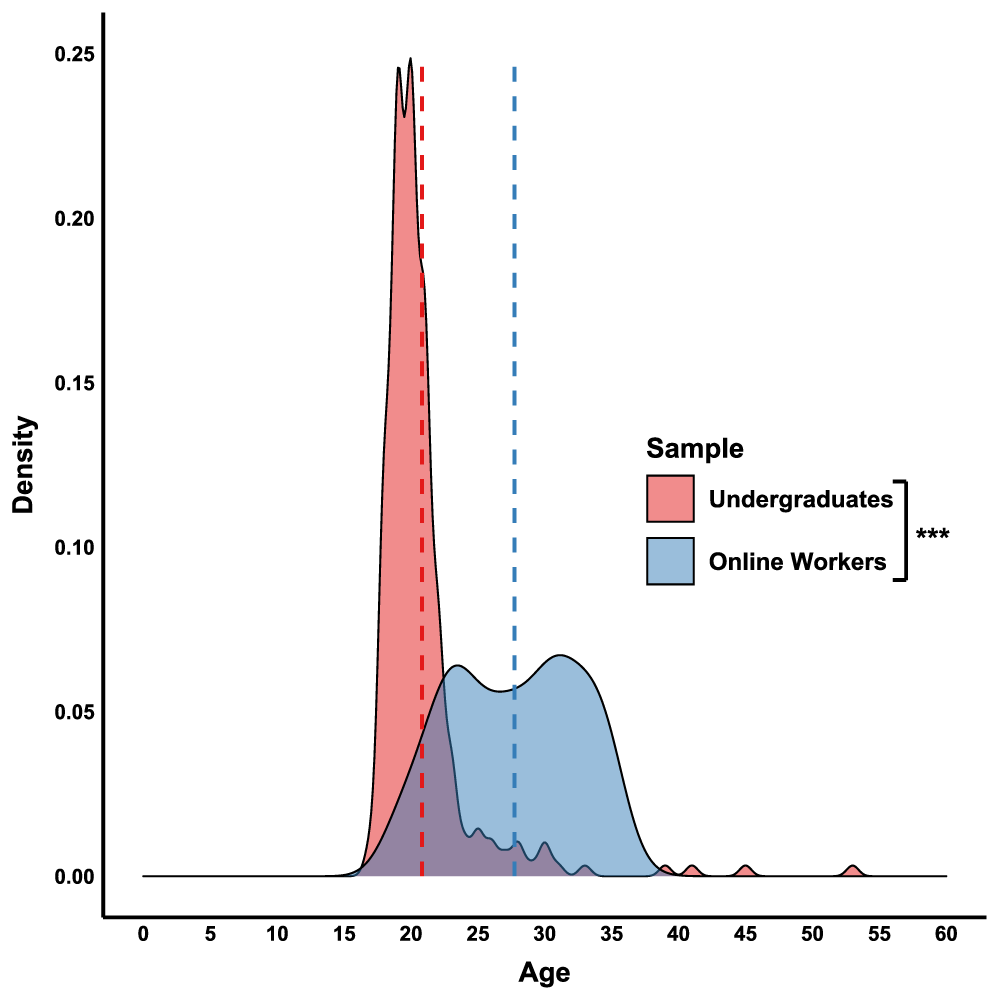

Supplement: Figure 2-2 — Study 1: Age Distribution Across Samples. Density plot representing the distribution of ages for undergraduates (red) and online workers (blue). Dashed vertical lines represent the mean age for each group. A significant difference in age between the samples are indicated (p < .001), with online workers being older on average compared to undergraduates. Download Figure 2-2, TIF file. [file eneuro-12-ENEURO.0034-25.2025-s007.tif]

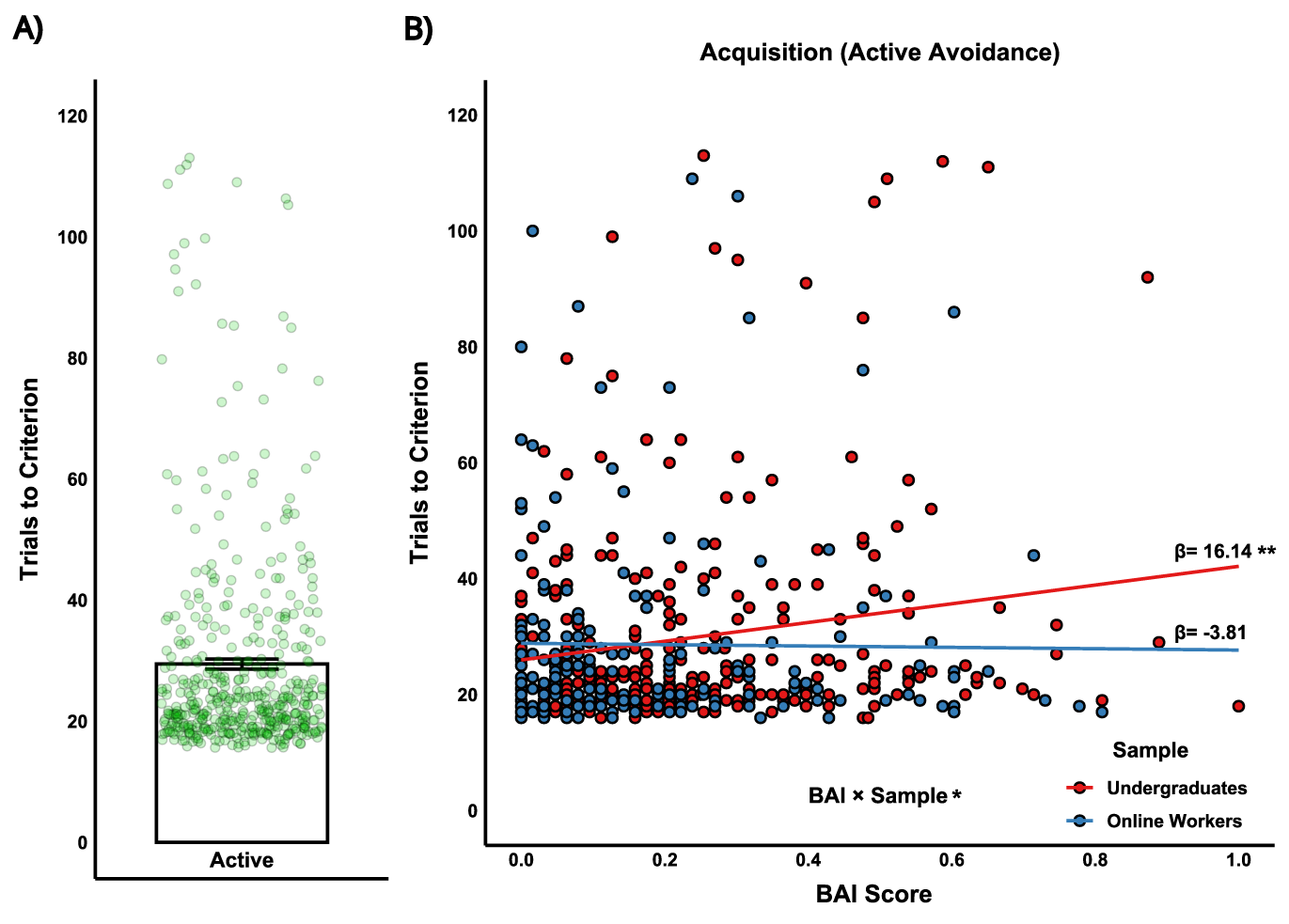

Supplement: Figure 4-1 — Study 1: Trials to Criterion for Active Avoidance During the Acquisition Stage. A) Number of trials required to reach criterion for all participants in the avoidance task. Individual data points (green circles) represent the number of trials each participant required to reach the criterion of 80% correct responses within 20-trial period during acquisition. B) Interaction between anxiety scores (BAI proportion scores) and the sample group (Undergraduates vs. Online Workers) predicting trials to criterion for active avoidance. The regression lines show the relationship between anxiety scores and trials to criterion for each sample, with a stronger effect observed in undergraduates (β = 16.14) compared to online workers (β = -3.81). A significant main effect of BAI scores (p < .01) and a significant BAI × Sample interaction (p < .05) are indicated. Download Figure 4-1, TIF file. [file eneuro-12-ENEURO.0034-25.2025-s008.tif]

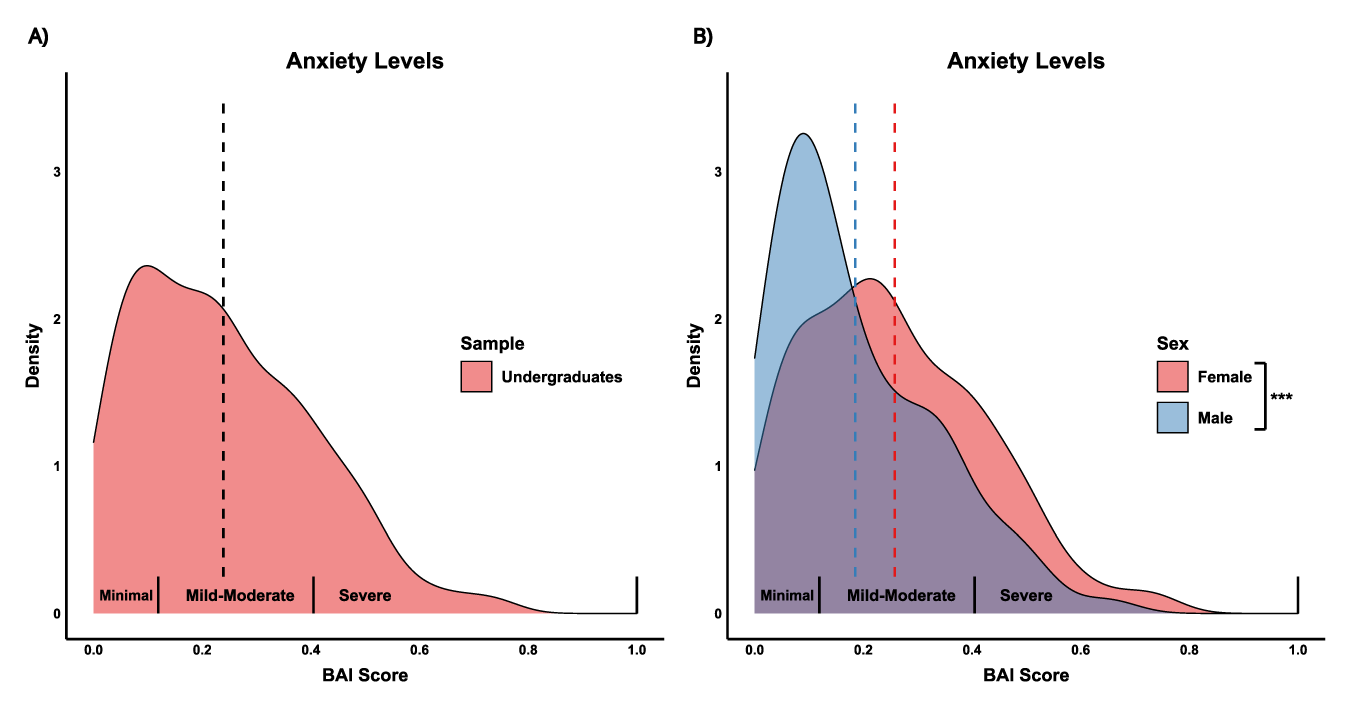

Supplement: Figure 7-1 — Study 2: Distribution of Anxiety Scores in Undergraduates and Across Sexes. Density plots representing the distribution of Beck Anxiety Inventory (BAI) scores. A) Anxiety score distributions in full undergraduate sample (red). B) Anxiety score distributions by sex: Female (red) and Male (blue). The x-axis represents proportion scores, where raw BAI scores, ranging from 0-63, have been divided by the maximum possible score (63) to produce a proportion between 0 and 1. This adjustment was made for comparability between the BDI-II and BAI scales. The labels on the x-axis -- Minimal (0-7), Mild-Moderate (8-25), Severe (26-63) -- reflect typical ranges of raw scores for ease of interpretation. Dashed vertical black line represents mean in full sample. Dashed vertical-coloured lines represent the mean BAI score for each sex. In panel B, a significant difference in anxiety levels between sexes is indicated (p < .001), with females scoring higher on average than males. Download Figure 7-1, TIF file. [file eneuro-12-ENEURO.0034-25.2025-s009.tif]

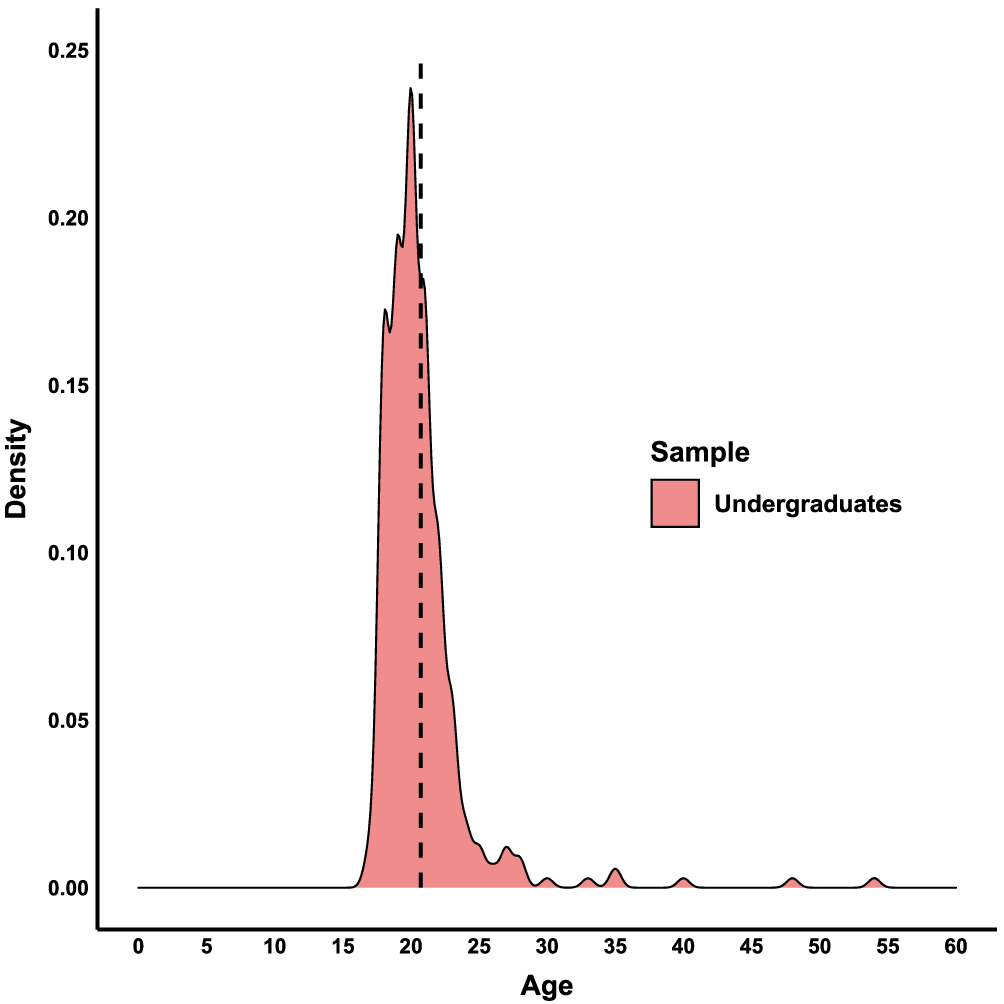

Supplement: Figure 7-2 — Study 2: Age Distribution in Undergraduates. Density plot representing the distribution of ages for undergraduates (red). Dashed vertical black line represent the mean age. Download Figure 7-2, TIF file. [file eneuro-12-ENEURO.0034-25.2025-s010.tif]
